# Synergism between Angiotensin receptors ligands: Role of Angiotensin‐(1‐7) in modulating AT_2_R agonist response on nitric oxide in kidney cells

**DOI:** 10.1002/prp2.667

**Published:** 2020-11-16

**Authors:** Sanket Patel, Tahir Hussain

**Affiliations:** ^1^ Department of Pharmacological and Pharmaceutical Sciences College of Pharmacy University of Houston Houston TX USA

**Keywords:** angiotensin receptor, epithelial cells, nitric oxide, receptor mas, synergism

## Abstract

Angiotensin‐(1‐7), an endogenous agonist for the MasR, has been shown to interact with ang‐II AT_1_R and AT_2_R. Earlier we showed a physical and functional interaction between MasR and AT_2_R in response to their respective agonists ang‐(1‐7) and C21. Moreover, ang‐(1‐7) is cardio‐protective via AT_1_R and alters ang‐II function. Such complex nature of ang‐(1‐7) function is not clearly understood, particularly in relation to its functional interaction with these receptors. We tested how ang‐(1‐7) affects AT_2_R function by utilizing HK‐2 cells. The HK‐2 cells were treated with a wide range of concentrations of angiotensin receptor agonists. The generation of NO^•^ in response to agonists was determined as a readout and subjected to Bliss definition (δ score) to assess the nature of functional interaction between these receptors. Preincubation with ang‐(1‐7) followed by incubation with endogenous AT_1_R/AT_2_R agonist ang‐II (δ = 162) or selective AT_2_R agonist C21 (δ = 304) synergized NO^•^ formation. The synergism was also observed when the order of incubation with ang‐(1‐7)/C21 was reversed (δ = 484), but not when the cells were simultaneously incubated with a mixture of ang‐(1‐7) and C21 (δ = 76). The synergism with nonpeptidic MasR agonist AVE0991 followed by C21 (δ = 45) was minimal. Ligand binding experiment suggested the binding of ang‐(1‐7) with these three receptors. However, the synergism observed with ang‐(1‐7) and ang‐II/C21 was sensitive to the antagonists of AT_2_R (PD123319) and AT_1_R (candesartan), but not MasR (A779). Ang‐(1‐7) at lower concentrations synergies the AT_2_R function in an AT_1_R‐dependent but MasR‐independent manner. This phenomenon may have a physiological significance.

## Graphical abstract

Synergism between ang‐(1‐7) and angiotensin‐II type 2 receptor ligands C21 and angiotensin‐II.

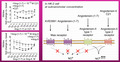

**Abbreviations**
Ang‐(1‐7): angiotensin‐(1‐7)
Ang‐II: angiotensin‐II
AT_1_R: angiotensin‐II type 1 receptor
AT_2_R: angiotensin‐II type 2 receptor
HK‐2: human kidney‐2 cells
MasR: receptor mas
RAS: renin‐angiotensin system

**What is already known**

- Endogenous MasR agonist and a major RAS peptide hormone, ang‐(1‐7) may act via AT_1_R or AT_2_R.
- Several functions of AT_1_R/AT_2_R are similar and AT_1_R and AT_2_R may act cooperatively.

**What this study adds**

- The functional screening approach employed in this study advances our understanding of synergistic interactions occurring at physiological concentrations among endogenous RAS peptides.
- The MasR agonist ang‐(1‐7) acts, in part, via AT_1_R and remarkably synergizes AT_2_R function of NO^•^ formation in kidney cells.

**What is the clinical significance**

- As angiotensin receptors have been implicated in myriad comorbid conditions such as obesity, hypertension, and kidney failure, the understanding of cooperative nature of angiotensin receptors and synergistic interactions among endogenous peptides, ang‐II and ang‐(1‐7) and pharmacological agonist such as C21 in NO^•^ formation is clinically relevant.

## 1 INTRODUCTION

Physical interactions, dimerization, and functional interdependency among receptors of the renin‐angiotensin system (RAS) is an evolving field. Angiotensin‐II (ang‐II), an octapeptide, is a key hormone of RAS that acts on angiotensin‐II type 1 (AT _1_ R) and type 2 (AT _2_ R) receptors, which belong to the G‐protein coupled receptor superfamily. The ang‐II signaling through AT_1_R triggers either canonical activation of G‐protein that accounts for most of the classical actions including sodium reabsorption or initiates G‐protein independent pathway.^1^, ^2^ The AT_1_R remains in an inactive state and requires an agonist binding for activation.^3^ On the other hand, AT_2_R has constitutively active conformation and is linked to nitric oxide formation.^3^, ^4^ Generally, AT_1_R and AT_2_R perform opposite functions,^5^, ^6^, ^7^ however, it has been reported that their signaling pathways may overlap.^8^, ^9^, ^10^, ^11^ For instance, ang‐II at physiological concentration promotes the dimerization of AT_1_R and AT_2_R and internalize as ang‐II‐AT_1_R/AT_2_R complex.^12^ AT_1_R and AT_2_R both facilitate ang‐II‐induced nitric oxide formation^13^ showing their cooperativity under physiological conditions.^12^, ^14^

Angiotensin‐(1‐7) (ang‐(1‐7)), a heptapeptide, is generally considered as an agonist of receptor Mas (MasR). However, numerous studies have reported that ang‐(1‐7) also mediates its responses via AT_1_R as well as AT_2_R. Ang‐(1‐7) binding to AT_1_R has been recently confirmed in AT_1_R‐transfected HEK293.^1^, ^2^ The functional roles of ang‐(1‐7) via these receptors are still unclear, particularly in terms of their physical and functional interaction. Based on a recent study, it is clear that unlike ang‐II, ang‐(1‐7) activation of AT_1_R is cardio‐protective via biased signaling.^1^, ^2^ We and others have shown that ang‐(1‐7) is pronatriuretic^15^ and antihypertensive.^16^ However, ang‐(1‐7)‐mediated natriuresis and nitric oxide production were blocked by the AT_2_R antagonist PD123319 as well as by the MasR antagonist A779.^15^ Similar observation on the antagonism of ang‐(1‐7) response by both the receptors antagonists was made in another study.^17^ Antinatriuretic response to ang‐(1‐7) has also been reported,^18^ probably due to its ability to stimulate Na‐ATPase in the proximal tubules,^19^ a phenomenon similar to ang‐II‐induced antinatriuresis and Na‐ATPase stimulation.^20^ Interestingly, the combined effect of ang‐(1‐7) and ang‐II on Na‐ATPase stimulation was either antagonistic^19^ or additive,^21^ depending on which of the peptides was used to preincubate the proximal tubules for the Na‐ATPase assay. Collectively, these studies led to the question of whether a potential functional interaction among angiotensin peptides on AT_1_R, AT_2_R_,_ and MasR exists. This study attempts to address this question by utilizing angiotensin peptides and specific agonists for AT_2_R and MasR over a wide range of concentrations and various combinations in human kidney proximal tubule epithelial cells (HK‐2 cells) grown in a 96‐well plate. Nitric oxide which has a wide range of physiological significance including natriuresis that is linked to all the three receptors (Table 1),^13^, ^15^, ^22^, ^23^, ^24^, ^25^ and was used as an end‐point functional readout.

**Table 1:** Ligands used in the study and their receptors

Ligand	Receptor
Angiotensin‐II	Angiotensin‐II type 1 receptor (AT_1_R); Angiotensin‐II type 2 receptor (AT_2_R)
Angiotensin‐(1‐7)	Mas receptor (MasR); Angiotensin‐II type 1 receptor (AT_1_R); Angiotensin‐II type 2 receptor (AT_2_R)
C21	Angiotensin‐II type 2 receptor (AT_2_R)
AVE0991	Mas receptor (MasR)

The synergy can be quantified through the use of a reference mathematical model. Although, according to Saariselka agreement, the best reference model to quantitate synergy and a recommendation or practical guideline to select a model over another do not exist,^26^ the Bliss definition of independence as a reference model (originally proposed in 1939 by Bliss CI) can be employed to assess the degree of synergism. Therefore, in this study the synergistic effects among various angiotensin receptor agonists utilizing nitric oxide as an end‐point was assessed as an effect‐based approach employing the Bliss definition of independence as a reference model (originally proposed in 1939 by Bliss CI) hypothesizing that the effects exerted by ligands studied are similar and independent,^26^, ^27^ ie, ligands may not act through a common receptor or a mechanism. We find that ang‐(1‐7) remarkably synergizes the AT_2_R functional response elicited by either ang‐II or C21 and this synergism was partly mediated via an AT_1_R‐dependent, but MasR‐independent mechanism.

## 2 MATERIALS AND METHODS

### 2.1 Cell culture

HK‐2 cells (CRL‐2190, ATCC) were cultured in DMEM/F‐12 media containing 10% heat‐inactivated fetal bovine serum, bovine pituitary extract (0.05 mg/ml), epidermal growth factor (5 ng/ml), and antibiotic‐antimycotic. All cell culture reagents were purchased from Thermo Fisher Scientific.

### 2.2 Treatment with agonists/antagonists

Cells (1X10^4^ per well) (passage 5‐15) were seeded onto a 96‐well plate. On the day of the experiment, the media was replaced with fresh DMEM/F‐12 free of phenol red, serum, growth factors, and antibiotics. A binary combination approach was used to screen six‐to‐eight concentrations of agonists to determine their factual functional interactions.

In preincubation experiments, cells were preincubated without or with various concentrations of ang‐(1‐7) (APExBIO Technology) (10^‐12^‐10^‐5^M) for 10 minutes, followed by addition of various concentrations of AT_1_R/AT_2_R agonist ang‐II (Sigma‐Aldrich) (10^‐12^‐10^‐5^M) or preferential AT_2_R agonists C21 (a gift from Vicore Pharma) (10^‐12^‐10^‐5^M), and incubation was continued in a cell culture incubator at 37°C and 5% CO_2_ for an additional 1 hour as described earlier.^12^, ^14^

In another experiment, the order of addition of agonists was reversed, ie, cells were preincubated without or with various concentrations of C21 (10^‐12^‐10^‐5^M) for 10 minutes, followed by the addition of various concentrations of ang‐(1‐7) (10^‐12^‐10^‐5^M) and incubation was continued as explained earlier.

In the coincubation experiment, a mixture of ang‐(1‐7) (10^‐10^‐10^‐5^M) and C21 (10^‐10^‐10^‐5^M) was added to the cells for 1 hour.

In another set of experiments, cells were preincubated without or with various concentrations of the nonpeptidic MasR agonist AVE0991 (AVE) (APExBIO Technology) (10^‐10^‐10^‐5^M) for 10 minutes, followed by the addition of various concentrations of C21 (10^‐10^‐10^‐5^M) and incubation was continued as explained earlier.

In the experiment with antagonists, cells were preincubated with the antagonists of AT_1_R (candesartan) (a gift from AstraZeneca), AT_2_R (PD123319) (Cayman Chemical) or MasR (A779) (Cayman Chemical) (all 10^‐5^M) for 15 minutes before incubation with ang‐(1‐7) followed by addition of ang‐II/C21 as explained earlier.

### 2.3 Measurement of total nitrites

The formation of nitric oxide, measured as total nitrites, was set as a functional readout and detected in media as total nitrites using Griess reagent as we have described earlier.^28^ Briefly, the cell culture supernatant (120 µl) was collected after agonists/antagonists treatment and transferred to another clear 96‐well plate and incubated with nitrate reductase enzyme (13 µl) (Cayman Chemical) and cofactor preparation (13 µl) (Cayman Chemical) for 2 hours at 37°C. Samples and nitrite standards (0‐25 µmole/l) (Cayman Chemical) were allowed to react with sulfanilamide (50 µl, 1% in 5% phosphoric acid) (TCI America) for 10 minutes on gentle shaking. The reaction was continued with the addition of N‐(1‐naphthyl)ethylenediamine dihydrochloride (50 µl, 0.1% in distilled water) (Sigma‐Aldrich). Absorbance was immediately read in Varioskan Flash plate reader (Thermo Fisher Scientific) at 540 nm. The total nitrites were normalized to basal and fold change values.

### 2.4 Analysis of synergy

The total nitrite values were transformed to percentages and the concentration‐response matrix was uploaded on SynergyFinder (version 2.0), an interactive stand‐alone web application for processing and scoring of synergy (δ);^27^, ^29^ higher the δ score, better is the synergistic response. Normally, the degree of synergy is quantified based on the comparison of the expected and the observed combination responses under the assumption that ligands being tested are acting independently or via a similar mechanism using an appropriate reference mathematical model. The SynergyFinder implements R‐based algorithms that compare the observed combination responses with expected responses and based on deviation of observed and expected responses, it classifies the combination as synergistic (ie, combination effect is higher than expected) or antagonistic (ie, combination effect is lower than expected). The R‐package and its source‐codes are freely available.^27^ The results were fitted with a four‐parametric nonlinear model (default option) without omitting outliers. We have used a well‐accepted, simple, yet stringent method, the Bliss definition of independence to determine whether functional interactions among agonists are synergistic or antagonistic. Specifically, the Bliss definition is a reference model that is formulated upon a null hypothesis and treats a drug combination as noninteracting. The Bliss independence model employs a probabilistic perspective and allows the expected combination response to be computed as the multiplicative product of individual drug response; whereas other models such as the Zero Interaction Potential (ZIP) model combines the Bliss model and the Loewe's additivity model. The Loewe's additivity model relies on the assumption that ligands are acting on the same target or through similar mechanism(s).^26^ Hence, we have used the Bliss definition of independence to analyze synergy with the intuition that ligands are acting through different receptors/mechanisms. The use of an interactive surface map over a full concentration matrix was used to readily visualize synergism or antagonism.

### 2.5 Ligand binding experiment

Binding experiment of 5(6)‐carboxyfluorescein (FAM)‐labeled ang‐(1‐7) (Phoenix Pharmaceuticals, Inc) was performed using live HK‐2 cells as previously described.^30^ Cells were seeded in glass‐bottom, black, 24‐well Sensoplate (VWR International). Competition binding was performed with FAM‐ang‐(1‐7) (10^‐9^ M) with or without unlabeled ang‐(1‐7), A779, candesartan, PD123319, ang‐II or C21 (all 10^‐6^ M) in a total of 0.5 ml DMEM/F‐12 supplemented with 0.1% bovine serum albumin, 0.1 mmol/L ortho‐phenanthroline and 0.5 mmol/L EDTA and free of phenol red, serum, growth factors, and antibiotics for 1 hour at 37⁰C in a cell culture incubator. Cells were gently washed three times with phosphate buffer saline and analyzed through Expression Analysis application and bright field segmentation algorithm of Nexcelom Celigo Cytometer. The resulting images were subjected to sequential gating (integrated and mean intensity and size) on the GFP channel for the identification of FAM‐ang‐(1‐7) labeled cells. The percentages of FAM‐ang‐(1‐7) labeled cells in the presence of unlabeled ang‐(1‐7) are considered nonspecific, which was subtracted from the total for the specific binding which was considered as 100% for comparison with other ligands.

### 2.6 Statistical analysis

Nitrite data are represented as mean ± SEM of fold changes compared to basal. The interactions were subjected to two‐way ANOVA followed by Tukey's multiple comparison test or one‐way ANOVA followed by Fisher's least significant difference test using GraphPad prism 6. Data were considered statistically significant at *P* ≤ .05 (n = 3‐16). The n value representing various combination(s) is provided in supplementary data (SI Table S1‐ S5). The 2‐D plots, the calculation and visualization reports of the Bliss (SI Figures S2‐S11) and the ZIP (SI Figs. S12‐S21) synergy score derived from SynergyFinder (version 2.0) are provided as supplementary information. Synergy was only considered when the functional interaction was statistically significant (*P* ≤ .05) as compared to the single‐agonist concentration‐response to avoid the random selection of false‐positive concentration combinations of ligands similar to the Highest Single Agent approach or the Combination Subthresholding approach.^31^

### 2.7 Data availability

The authors declare that all data supporting the findings of this study are presented within the paper and are available from the authors upon request.

### 2.8 Nomenclature of targets and ligands

Key protein targets and ligands in this article are hyperlinked to corresponding entries in http://www.guidetopharmacology.org, the common portal for data from the IUPHAR/BPS Guide to PHARMACOLOGY,^32^ and are permanently archived in the Concise Guide to PHARMACOLOGY 2019/20.^33^

## 3 RESULTS

### 3.1 Synergistic formation of nitric oxide upon pre‐incubation of HK‐2 cells with ang‐(1‐7) followed by ang‐II

**Figure 1:**
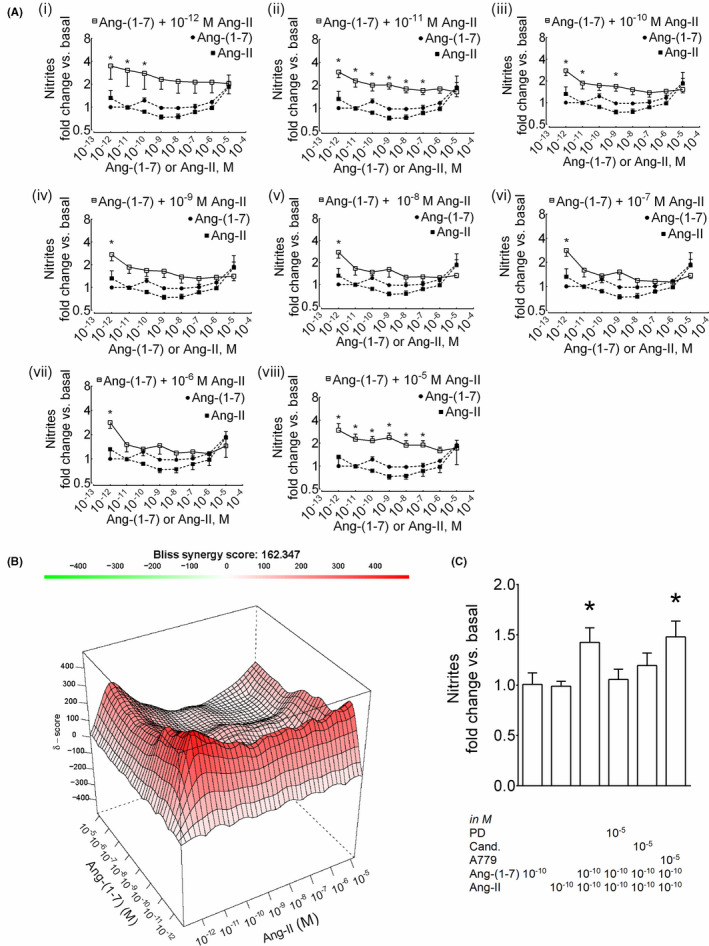
Synergistic effect of ang‐(1‐7) preincubation on NO^•^ response to AT_1_R/AT_2_R agonist ang‐II in HK‐2 cells. (A) Cells were preincubated with ang‐(1‐7) (10^‐12^‐10^‐5^ M) for 10 min followed by incubation with ang‐II (10^‐12^‐10^‐5^ M) for another 1 h (i‐viii). The data points of ligand combinations and single ligands are shown with open (□) and closed (●, ang‐(1‐7); ■, ang‐II) symbols, respectively. Results are mean ± SEM; *P < .05 vs. single‐agonist concentration‐response based on two‐way ANOVA followed by Tukey's posthoc test (n = 3‐11). (B) Surface plots show the concentration‐response matrix and overall Bliss energy score (δ = 162). (C) Effect of the antagonist of AT_2_R (PD123319), MasR (A‐779), and AT_1_R (candesartan) on NO^•^ response in HK‐2 cells. Cells were incubated with PD123319, A779, or candesartan (all 10^‐5^ M) for 10 minutes followed by incubation with ang‐(1‐7) (10^‐10^ M) for 10 min, then incubation with the AT_2_R agonist C21 (10^‐10^ M) for another 1 h. Results are mean ± SEM; *P < .05 vs. single agonist based on one‐way ANOVA followed by Fisher's Least Significance Difference test (n = 6‐7)

The interactions were synergistic when cells were preincubated with ang‐(1‐7) followed by ang‐II over the considerable region of a concentration‐response matrix showing maximal combination effect approximately 3‐4‐fold increase in nitrites formation (Figure 1A). The overall δ score for the combination matrix was 162 (Figure 1B) suggesting synergism. However, significant synergism was observed at the lowest concentration of ang‐(1‐7) (10^‐12^ M) and all concentrations of ang‐II tested (10^‐12^‐10^‐5^ M) (Figure 1A), at various lower concentrations of ang‐(1‐7) (10^‐11^‐10^‐7^ M) and ang‐II (10^‐11^ M, Figure 1A(ii); 10^‐5^ M, Figure 1A(viii)). The synergistic interaction between ang‐(1‐7) and ang‐II was sensitive to antagonists of AT_2_R (PD123319) and AT_1_R (candesartan), but not MasR (A779) (Figure 1C). The effect of single ligand alone is also provided in SI Figure 1.

### 3.2 Synergistic formation of nitric oxide upon preincubation of HK‐2 cells with ang‐(1‐7) followed by C21

**Figure 2:**
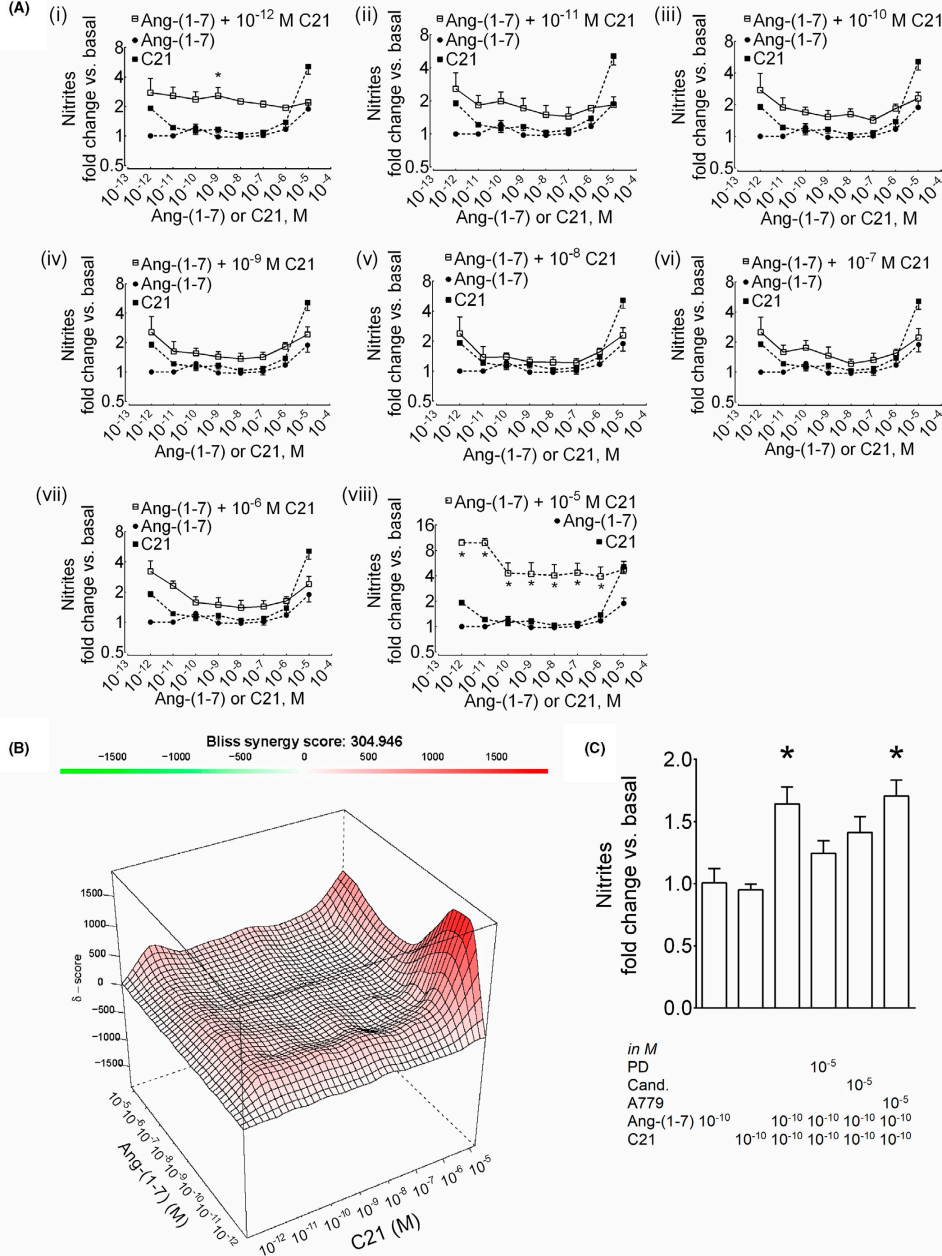
Synergistic effect of ang‐(1‐7) preincubation on NO^•^ response to AT_2_R agonist C21 in HK‐2 cells. (A) Cells were preincubated with ang‐(1‐7) (10^‐12^‐10^‐5^ M) for 10 minutes followed by incubation with C21 (10^‐12^‐10^‐5^ M) for another 1 h (i‐viii). The data points of ligand combinations and single ligands are shown with open (□) and closed (●, ang‐(1‐7); ■, C21) symbols, respectively. Results are mean ± SEM; *P < .05 vs. single‐agonist concentration‐response based on two‐way ANOVA followed by Tukey's posthoc test (n = 3‐11). (B) Surface plots show the concentration‐response matrix and overall Bliss energy score (δ = 304). (C) Effect of the antagonist of AT_2_R (PD123319), MasR (A‐779), and AT_1_R (candesartan) on NO^•^ response in HK‐2 cells. Cells were incubated with PD123319, A779, or candesartan (all 10^‐5^ M) for 10 min followed by incubation with ang‐(1‐7) (10^‐10^ M) for 10 min, then incubation with the AT_2_R agonist C21 (10^‐10^ M) for another 1 h. Results are mean ± SEM; *P < .05 based on one‐way ANOVA followed by Fisher's Least Significance Difference test (n = 6‐16)

The nitrite responses in HK‐2 cells after preincubation with ang‐(1‐7) and C21 were synergistic over a range of ang‐(1‐7) concentrations (10^‐12^‐10^‐7^ M) (Figure 2A(i)) (Figure 2A(viii)). Specifically, the observed synergism was maximum (~8 fold) at the lowest concentration of ang‐(1‐7) (10^‐12^‐10^‐11^ M) (Figure 2A(viii)) (Figure 2B). Surprisingly, the degree of synergism was decreased upon increasing ang‐(1‐7) concentration (Figure 2A(viii)). The synergism between ang‐(1‐7) and C21 also remained sensitive to the AT_2_R antagonist PD123319 and the AT_1_R antagonist candesartan, but not to the MasR antagonist A779 (Figure 2C).

### 3.3 Increase in synergistic formation of nitric oxide while order of addition is reversed; preincubation of HK‐2 cells with C21 followed by ang‐(1‐7)

**Figure 3:**
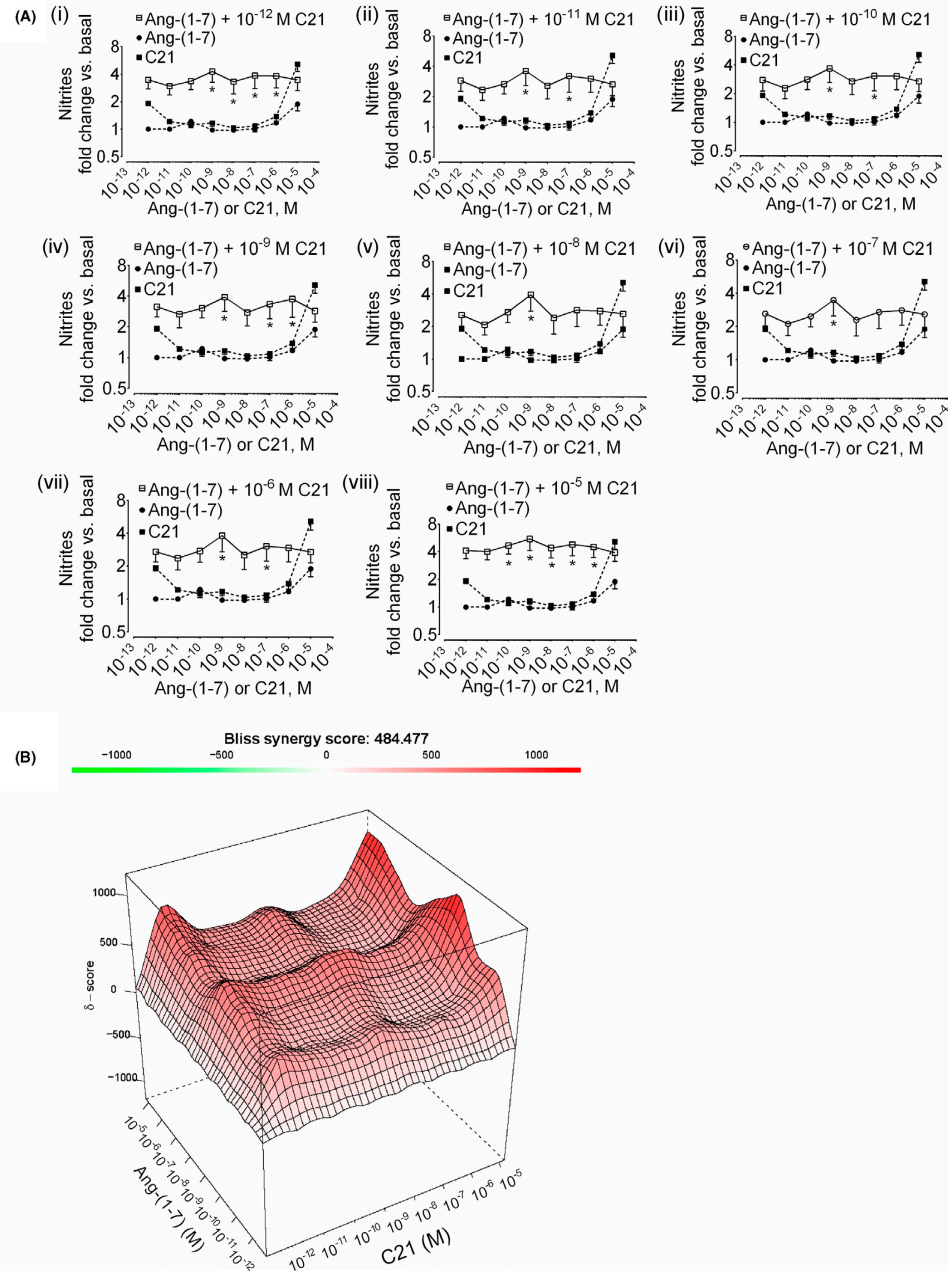
Synergistic effect of C21 preincubation on NO^•^ response to ang‐(1‐7) in HK‐2 cells. (A) Cells were preincubated with C21 (10^‐12^‐10^‐5^ M) for 10 min followed by incubation with ang‐(1‐7) (10^‐12^‐10^‐5^ M) for another 1 hour (i‐viii). The data points of ligands combinations and single ligands are shown with open (□) and closed (●, ang‐(1‐7); ■, C21) symbols, respectively. Results are mean ± SEM; *P < .05 vs. single‐agonist concentration‐response based on two‐way ANOVA followed by Tukey's posthoc test (n = 3‐12). (B) Surface plots show the concentration‐response matrix and overall Bliss energy score (δ = 484)

The order of addition of agonists was reversed to determine whether preincubation with ang‐(1‐7) is a prerequisite to observe synergistic nitrite response to the AT_2_R agonist C21. C21 was chosen over ang‐II as synergy score with C21 experiments (δ = 304, Figure 2B) was better as compared to that of ang‐II (δ = 162, Figure 1B). The nitrite responses with C21 preincubation followed by addition of ang‐(1‐7) universally remained high at all concentrations tested (Figure 3A) with improved synergy score (δ = 484) (Figure 3B).

### 3.4 Decrease in synergism of nitric oxide formation upon incubation of HK‐2 cells with a mixture of ang‐(1‐7) and C21

**Figure 4:**
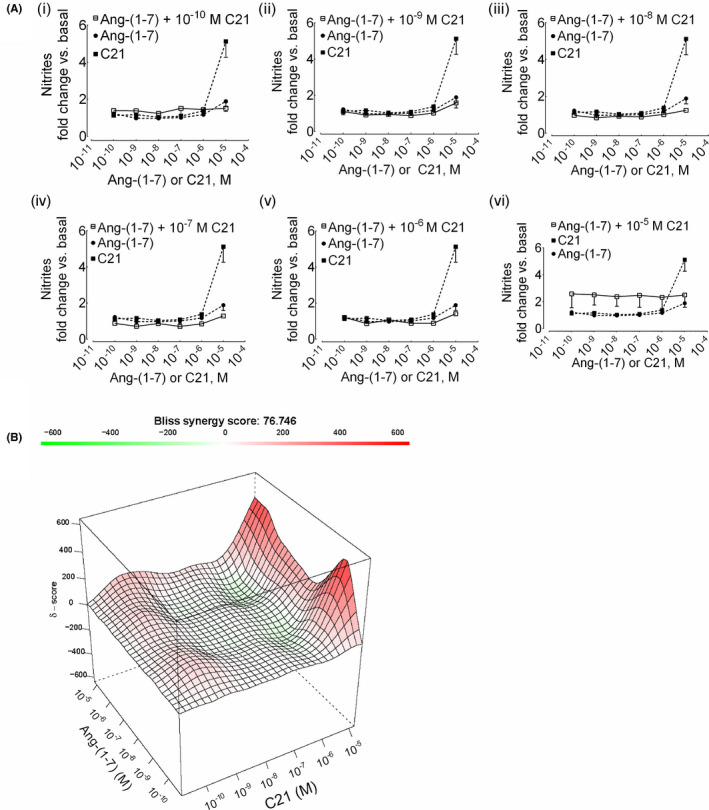
Reduction in synergy upon incubation of HK‐2 cells with a mixture of ang‐(1‐7) and C21. (A) Cells were incubated with a mixture of various concentrations of ang‐(1‐7) and C21 (10^‐10^‐10^‐5^ M) for 1 hour (i‐vi). The data points of ligands combinations and single ligands are shown with open (□) and closed (●, ang‐(1‐7); ■, C21) symbols, respectively. Results are mean ± SEM (n = 3‐12). (B) Surface plots show the concentration‐response matrix and overall Bliss energy score (δ = 76)

We attempted to determine whether it is imperative to incubate cells with agonists sequentially to observe synergistic nitrite response, we incubated cells with a mixture of ang‐(1‐7) and C21. Surprisingly, the mixture (agonists added together) of ang‐(1‐7) and C21 at any combinations tested did not result in a significant formation of nitrites as compared to that of single‐agonists alone and the overall synergy dropped tremendously (δ = 76) (Figure 4).

### 3.5 Decrease in synergism of nitric oxide formation upon incubation of HK‐2 cells with AVE0991 followed by C21

**Figure 5:**
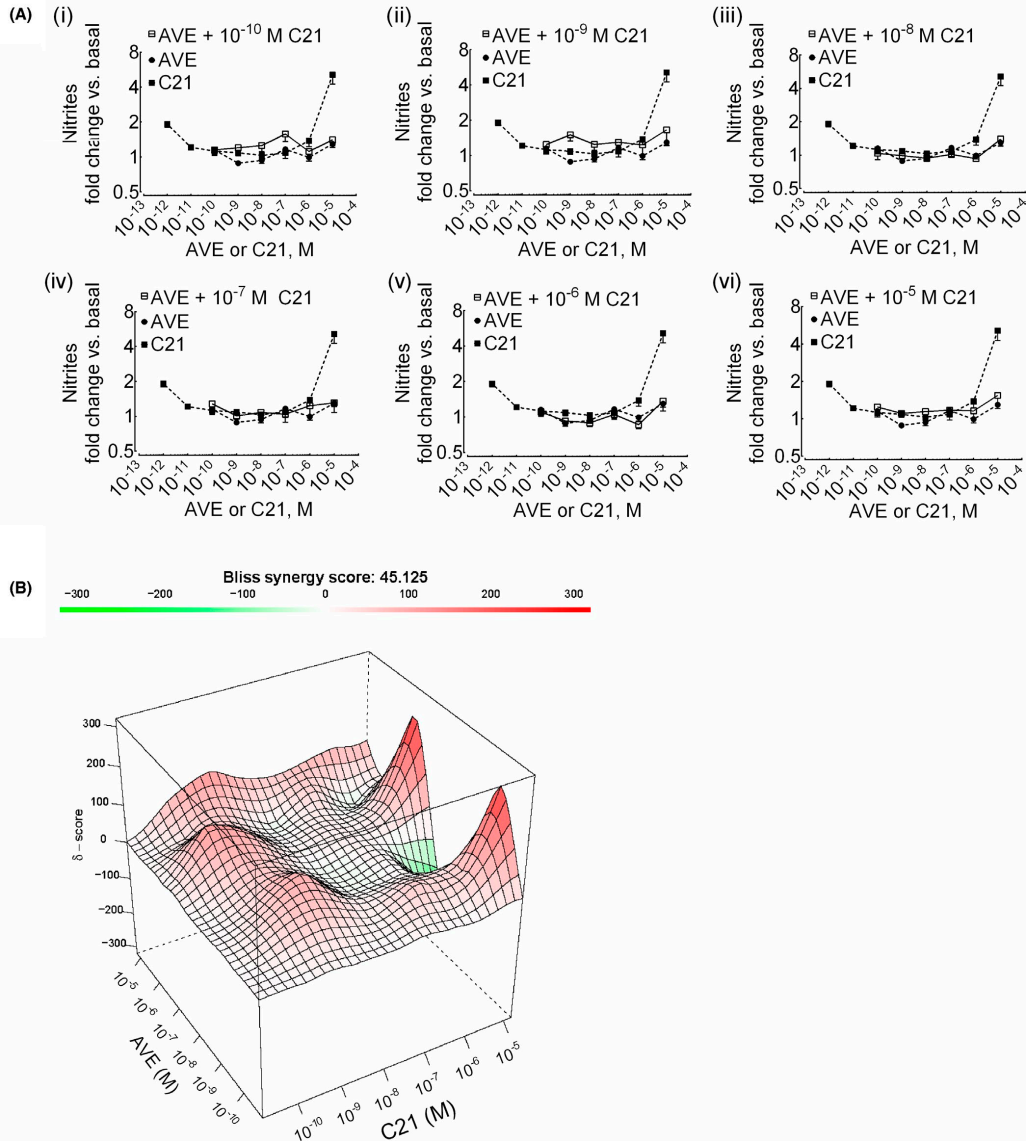
Reduction in synergy upon preincubation with nonpeptidic MasR agonist AVE0991 (AVE) followed by incubation with AT_2_R agonist C21 in HK‐2 cells. (A) Cells were pre‐incubated with AVE (10^‐10^‐10^‐5^ M) for 10 minutes followed by incubation with C21 (10^‐10^‐10^‐5^ M) for another 1 hour (i‐vi). The data points of ligand combinations and single ligands are shown with open (□) and closed (●, AVE; ■, C21) symbols, respectively. Results are mean ± SEM (n = 3‐12). (B) Surface plots show the concentration‐response matrix and overall Bliss energy score (δ = 45)

To determine whether the observed synergistic phenomenon is specific to ang‐(1‐7), we preincubated cells with various concentrations of the nonpeptidic MasR preferential agonist AVE0991 followed by the addition of various concentrations of C21. The nitrite responses of combinations of AVE0991 and C21 were not statistically different as compared to their effects alone (Figure 5A) with a dampened synergy score (δ) of 45 (Figure 5B).

### 3.6 Displacement of FAM‐labeled ang‐(1‐7)‐specific binding by ligands of AT1R, AT2R and MasR

**Figure 6:**
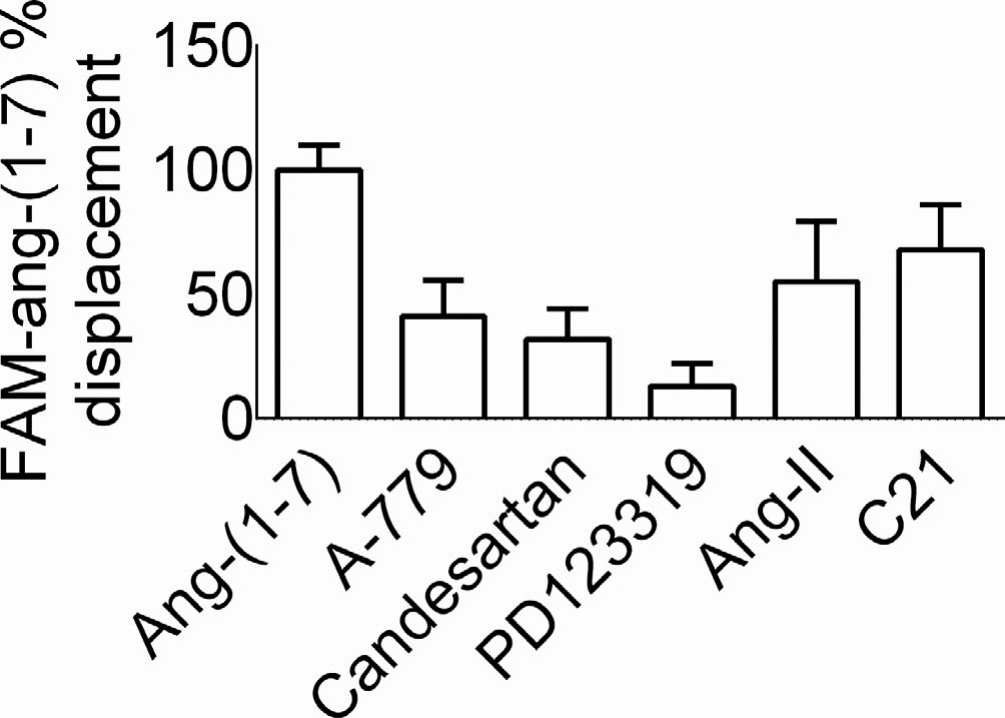
Competition binding experiment of 5(6)‐carboxyfluorescein (FAM)‐labeled ang‐(1‐7) in HK‐2 cells. Cells were seeded in glass‐bottom, black, 24‐well Sensoplate. Competition binding was carried out with FAM‐ang‐(1‐7) (10^‐9^ M) without or with unlabeled ang‐(1‐7), A779, candesartan, PD123319, ang‐II or C21 (all 10^‐6^ M). Cells were washed with PBS and analyzed through Expression Analysis application and bright field segmentation algorithm of Nexcelom Celigo Cytometer. The resulting images were subjected to sequential gating (integrated and mean intensity and size) on the GFP channel for the identification of FAM‐ang‐(1‐7) labeled cells. The percentages of FAM‐ang‐(1‐7) labeled cells in the presence of unlabeled ang‐(1‐7) are considered nonspecific, which was subtracted from the total for the specific binding which was considered as 100% for comparison with other ligands. Results are mean ± SEM (n = 4‐7)

The competition binding data shown in Figure 6 revealed that the MasR antagonist A799 and the AT_1_R antagonist candesartan displaced approximately 50% and 40%, respectively, of the FAM‐labeled ang‐(1‐7)‐specific binding while the AT_2_R antagonist PD123319 displaced only 10% of the specific binding. Ang‐II and C21 caused more than 50% displacement of the FAM‐labeled ang‐(1‐7) binding.

## 4 DISCUSSION

The ang‐(1‐7) is a biologically active major peptide hormone of RAS and putative MasR agonist but may also act via angiotensin receptors (AT_1_R, AT_2_R). These three receptors are shown to form functional homo‐/hetero‐dimers or oligomers^12^, ^14^, ^15^, ^17^, ^34^, ^35^, ^36^ and share NO^•^ as a common signaling molecule.^13^ The expression of MasR, AT_1_R, and AT_2_R^36^, ^37^ and other RAS components ^38^ in HK‐2 cells have been reported. Considering these, we attempted to determine that a positive modulatory interaction is plausible among these receptors with a functional consequence in terms of the second messenger NO^•^ formation at the cellular level. While the sub‐nM and ‐pM concentrations of these angiotensin peptides and C21 do not affect NO^•^ productions, only the combinations of ang‐(1‐7) with AT_1_R/AT_2_R agonist ang‐II or AT_2_R agonist C21, at picomolar concentrations, are highly synergistic in NO^•^ formation. In light of the kidney concentration (sub‐nM) of these peptides and even lower concentrations (pM) in extracellular space,^39^, ^40^, ^41^ our findings showing a strong synergistic interaction among these peptides may have a physiological significance. The Bliss definition of independence combined with statistical analysis reveals that the concentration of either agonist can be reduced by thousand‐fold in vitro. The observed synergy was also compared using the ZIP reference model that combines the Bliss independence and the Loewe's additivity models. The Loewe's additivity model relies on the assumption that ligands act in a similar manner, ie, through same target/mechanism. And, we have observed similar trend in synergy scores, ie, Bliss synergy vs. ZIP synergy (SI Table S6) for different ligand incubation approaches studied, although with decreased ZIP synergy scores (SI Figs. S12‐S21). Moreover, the ZIP synergy score was unable to distinguish the differences in the synergy observed with different incubation approaches. Hence, we adhered to Bliss definition and it is intuitive to believe that ligands rather are acting in an independent manner. Therefore, we rationalize that preincubation with ang‐(1‐7) may occupy AT_1_R and thus may allow the subsequently added AT_2_R ligand ang‐II or C21 to act upon AT_2_R, ie, involvement of different targets. We also have analyzed some of the results by employing Chou and Talalay's theorem based on effect size (Fa), the combination index (CI) and the dose reduction index (DRI) and found that the interactions are synergistic upon preincubation of HK‐2 cells with ang‐(1‐7) followed by C21 (SI Fig. S22‐S23).^42^, ^43^

Fundamentally, interactions between agonists may not be linear, ie, synergism may exist for a specific pair of concentrations and not for an entire range of concentrations.^44^ For this synergism, it was important that the agonists were added sequentially, (a) preincubation of HK‐2 cells with ang‐(1‐7) followed by addition of ang‐II or C21, or (b) when the order of addition was reversed, ie, preincubation of HK‐2 cells with C21 followed by addition of ang‐(1‐7). Because when the agonists were added simultaneously to the cells the synergism was not observed. This suggests that prior incubation of ang‐(1‐7) is not a requirement to observe the synergism. Notably, the degree of synergism observed is maximum at a lower range of concentrations of one agonist with either lower range or the highest concentration of another agonist. Reasons for synergism at the lower concentrations only and not at higher concentrations are not clear. However, some of the reasons could be that ang‐(1‐7)/ang‐II produce biphasic effects,^16^, ^19^, ^45^ ang‐II causes dissociation of AT_1_R‐AT_2_R heteromer,^46^ or internalization and desensitization of these receptors.^12^, ^45^ Moreover, based on competition ligand binding results, it appears that ang‐II/C21 could occupy almost 50% of the ang‐(1‐7) binding sites. An earlier study also has shown that ang‐(1‐7) displace high‐affinity ang‐II binding sites in rat glomeruli^47^ and functions of ang‐(1‐7) at the picomolar range were sensitive to AT_1_R antagonist supporting their interactions at high‐affinity site.^18^, ^48^, ^49^ Thus it's likely that the higher concentrations of these ligands may not show synergism due to a competition or steric hindrance^50^ to occupy receptor sites that perhaps overlap.

Antagonist displacement of ang‐(1‐7) data in this study appears to be a bit complicated but supported by previous studies showing interactions of ang‐(1‐7) via MasR, AT_1_R, and AT_2_R.^47^, ^48^ As expected MasR antagonist A779 and AT_1_R antagonist candesartan displaced ang‐(1‐7) but the AT_2_R antagonist PD123319 did not compete with the ang‐(1‐7) binding site, yet PD123319 completely antagonized the synergistic effects among ang‐(1‐7) and ang‐II or C21, most likely by blocking AT_2_R. This also suggests that PD123319 may also bind at different sites on AT_2_R as compared to those of ang‐II^47^ or ang‐(1‐7), and PD123319 may act via distinct unresolved AT_2_R‐related mechanism (vs. AT_1_R antagonists) in regulating ang‐(1‐7) effects.^51^ An early autoradiographic study using a nonselective AT_1_R and AT_2_R ligand ^125^I‐Sar^1^, Ile^8^ ang‐II did not show renal expression of the AT_2_R, while the autoradiography with the specific AT_2_R agonist ^125^I‐CGP42112b did show the presence of renal AT_2_R.^52^ The binding and functional mechanism related to the antagonists of these receptors is not fully understood. For instance, in our studies ang‐(1‐7) and ang‐II/C21 response was blocked by both the AT_1_R antagonist candesartan and the AT_2_R antagonist PD123319, while the ang‐II‐induced formation of NO^•^ in endothelial cells was blocked by the AT_1_R antagonist losartan, but not by PD123319.^25^ In another study, the ang‐II effect on calcium mobilization was blocked by both AT_1_R antagonist losartan and AT_2_R antagonist PD123319, but not when both antagonists were tested together suggesting their complex nature of binding to their receptors.^12^

The synergistic responses of ang‐(1‐7) with the endogenous AT_1_R/AT_2_R agonist ang‐II were wide‐spread over the lower range of concentrations as compared to that of ang‐(1‐7) with the AT_2_R preferential agonist C21 suggesting equal participation of AT_1_R and AT_2_R at lower concentrations. However, the observed synergy was higher and wide‐spread with combinations of higher concentrations of C21 and in experiments when cells were incubated with C21 before the addition of ang‐(1‐7) suggesting the dominance of AT_2_R at higher concentrations in synergy. Interestingly, the synergism was severely reduced when cells were preincubated with another nonpeptidic synthetic MasR agonist AVE0991 followed by AT_2_R agonist C21 indicating the involvement of different targets, ie, AVE0991 interacts with MasR while ang‐(1‐7) interacts with AT_1_R, MasR, and AT_2_R. This finding further supports that the observed synergism with ang‐(1‐7) and ang‐II/C21 involves AT_1_R and AT_2_R, but not MasR. We have reported the existence of MasR in HK‐2 cells and validated using siRNA knockdown experiment.^15^, ^37^ The displacement of ang‐(1‐7)‐specific binding by A779 also suggests the presence of MasR in HK‐2 cells. Collectively this study clearly suggests an interaction among naturally expressing angiotensin receptors in kidney cells leading to a functional synergism in response to lower concentrations of the agonists. However, further studies are required to reaffirm above notions and for a clear mechanistic understanding as to how agonists binding affects the conformation and activation of angiotensin receptor(s) or whether agonists bind to and/or activate monomers or hetero(oligomers) is yet to emerge.^36^ Regarding experiments performed in naturally expressing cells, as in our studies, it is difficult to deduce whether cellular response involves monomers, dimers, or (hetero)oligomers because receptors can integrate signals received by ligands via allosteric interaction within receptor‐receptor complexes. Receptor transfection/expression studies and receptor crystal structures may serve as potential tools that can advance our understanding of these GPCRs intermolecular interactions and of the diversity of the binding sites for various ligands that exert diverse and sometimes unexplainable functions. Moreover, this work relies on the assessment of nitric oxide a common signaling mediator of these receptors, in HK‐2 cell culture supernatant; how these combinations and the order of exposure of these ligands modulate other signaling molecules is not known. Hence, the findings are limited as we approached to determine nitric oxide formation as a sole outcome measure in cell culture supernatant. Further studies are required to determine whether such synergism exists in vivo and what physiological relevance these findings would have. Also, the approach to quantify the synergy does not allow us to predict the mode of action is a limitation.

The crosstalk among angiotensin receptors has recently been a subject of considerable debate. The ang‐II and ang‐(1‐7), major RAS peptide hormones acting via angiotensin receptors have been reported to play a crucial role in a plethora of physiological conditions such as water and electrolyte balance, natriuresis and vasodilation.^53^ This work shows the involvement of AT_1_R in ang‐(1‐7)‐mediated synergism of AT_2_R function of NO^•^ formation expands our understanding of the cooperative nature of these receptors.

## ETHICS APPROVAL STATEMENT

This study does not include any study with animals or human participants.

## PATIENT CONSENT STATEMENT

Informed consent for this type of study is not required.

## PERMISSION TO REPRODUCE MATERIAL FROM OTHER SOURCES

This study did not reproduce any material without permission.

## CLINICAL TRIAL REGISTRATION

This study does not involve clinical trial.

## CONFLICT OF INTEREST

The authors declare no conflicts of interest.

## AUTHOR CONTRIBUTIONS

In consultation with TH, SP initiated the project, designed in vitro experiments, prepared the manuscript draft and TH gave feedback and edited the manuscript. SP and TH approved the final version of the manuscript.

## Supporting information

Tables S1‐S6, Figures S1‐S23
