# Supplementary material for: Synergism between Angiotensin receptors ligands: Role of Angiotensin‐(1‐7) in modulating AT2R agonist response on nitric oxide in kidney cells
Source: Pharmacol Res Perspect. 2020 Nov 16;8(6):e00667. doi: 10.1002/prp2.667 (PMC7668194; doi:10.1002/prp2.667)
Supplement: Supplementary file 1 — Tables S1‐S6, Figures S1‐S23 [file PRP2-8-e00667-s001.docx]

**Synergism between Angiotensin Receptors Ligands: Role of Angiotensin‐(1‐7) in Modulating AT_2_R Agonist Response on Nitric Oxide in Kidney Cells**

Sanket Patel and Tahir Hussain

Department of Pharmacological and Pharmaceutical Sciences, College of Pharmacy, University of Houston, Houston, Texas 77204, USA

**Running title:** synergism among angiotensin receptor ligands

**Correspondence:**

Tahir Hussain, PhD

Professor

Health Bldg. 2

College of Pharmacy

University of Houston

4849 Calhoun, Houston, Texas 77004, USA

E-mail: [thussain@central.uh.edu](mailto:thussain@central.uh.edu)

Ph.: +1-713-743-1276

Fax: +1-713-743-1884

**Supplementary information**

**Table 1.** Sample size (n) corresponding to the ligand combinations studied (as in Figure 1 of manuscript).

| Ang-(1-7) | | | | | | | | | |
| --- | --- | --- | --- | --- | --- | --- | --- | --- | --- |
| Ang-II | 0 M | 10^-12^ M | 10^-11^ M | 10^-10^ M | 10^-9^ M | 10^-8^ M | 10^-7^ M | 10^-6^ M | 10^-5^ M |
| 0 M |  | 3 | 3 | 6 | 6 | 6 | 8 | 11 | 11 |
| 10^-12^ M | 3 | 4 | 4 | 4 | 4 | 4 | 4 | 4 | 4 |
| 10^-11^ M | 6 | 4 | 7 | 7 | 7 | 7 | 7 | 7 | 4 |
| 10^-10^ M | 6 | 4 | 7 | 7 | 7 | 7 | 7 | 7 | 4 |
| 10^-9^ M | 6 | 4 | 7 | 7 | 7 | 7 | 7 | 7 | 4 |
| 10^-8^ M | 6 | 4 | 7 | 7 | 7 | 7 | 7 | 7 | 4 |
| 10^-7^ M | 6 | 4 | 7 | 7 | 7 | 7 | 7 | 7 | 4 |
| 10^-6^ M | 6 | 4 | 7 | 7 | 7 | 7 | 7 | 7 | 4 |
| 10^-5^ M | 3 | 4 | 4 | 4 | 4 | 4 | 4 | 4 | 4 |

Note: HK-2 cells were pre-incubated with ang-(1-7) followed by ang-II.

**Table 2.** Sample size (n) corresponding to the ligand combinations studied (as in Figure 2 of manuscript).

| Ang-(1-7) | | | | | | | | | |
| --- | --- | --- | --- | --- | --- | --- | --- | --- | --- |
| C21 | 0 M | 10^-12^ M | 10^-11^ M | 10^-10^ M | 10^-9^ M | 10^-8^ M | 10^-7^ M | 10^-6^ M | 10^-5^ M |
| 0 M |  | 3 | 3 | 6 | 6 | 6 | 8 | 11 | 11 |
| 10^-12^ M | 3 | 3 | 3 | 3 | 3 | 3 | 3 | 3 | 3 |
| 10^-11^ M | 3 | 3 | 3 | 3 | 3 | 3 | 3 | 3 | 3 |
| 10^-10^ M | 6 | 3 | 3 | 8 | 7 | 7 | 7 | 7 | 7 |
| 10^-9^ M | 6 | 3 | 3 | 8 | 7 | 7 | 7 | 7 | 7 |
| 10^-8^ M | 6 | 3 | 3 | 8 | 7 | 7 | 7 | 7 | 7 |
| 10^-7^ M | 8 | 3 | 3 | 8 | 7 | 7 | 7 | 7 | 7 |
| 10^-6^ M | 11 | 3 | 3 | 8 | 7 | 7 | 7 | 7 | 7 |
| 10^-5^ M | 11 | 3 | 3 | 8 | 8 | 8 | 8 | 8 | 8 |

Note: HK-2 cells were pre-incubated with ang-(1-7) followed by C21.

**Table 3.** Sample size (n) corresponding to the ligand combinations studied (as in Figure 3 of manuscript).

| Ang-(1-7) | | | | | | | | | |
| --- | --- | --- | --- | --- | --- | --- | --- | --- | --- |
| C21 | 0 M | 10^-12^ M | 10^-11^ M | 10^-10^ M | 10^-9^ M | 10^-8^ M | 10^-7^ M | 10^-6^ M | 10^-5^ M |
| 0 M |  | 3 | 3 | 6 | 6 | 6 | 8 | 11 | 11 |
| 10^-12^ M | 3 | 6 | 6 | 6 | 6 | 6 | 6 | 6 | 6 |
| 10^-11^ M | 3 | 6 | 6 | 6 | 6 | 6 | 6 | 6 | 6 |
| 10^-10^ M | 6 | 6 | 6 | 6 | 6 | 6 | 6 | 6 | 6 |
| 10^-9^ M | 6 | 6 | 6 | 6 | 6 | 6 | 6 | 6 | 6 |
| 10^-8^ M | 6 | 6 | 6 | 6 | 6 | 6 | 6 | 6 | 6 |
| 10^-7^ M | 8 | 6 | 6 | 6 | 6 | 6 | 6 | 6 | 6 |
| 10^-6^ M | 12 | 6 | 6 | 6 | 6 | 6 | 6 | 6 | 6 |
| 10^-5^ M | 12 | 6 | 6 | 6 | 6 | 6 | 6 | 6 | 6 |

Note: HK-2 cells were pre-incubated with C21 followed by ang-(1-7).

**Table 4.** Sample size (n) corresponding to the ligand combinations studied (as in Figure 4 of manuscript).

| Ang-(1-7) | | | | | | | |
| --- | --- | --- | --- | --- | --- | --- | --- |
| C21 | 0 M | 10^-10^ M | 10^-9^ M | 10^-8^ M | 10^-7^ M | 10^-6^ M | 10^-5^ M |
| 0 M |  | 6 | 6 | 6 | 8 | 11 | 11 |
| 10^-10^ M | 6 | 3 | 3 | 3 | 3 | 3 | 3 |
| 10^-9^ M | 6 | 3 | 3 | 3 | 3 | 3 | 3 |
| 10^-8^ M | 6 | 3 | 3 | 3 | 3 | 3 | 3 |
| 10^-7^ M | 8 | 3 | 3 | 3 | 3 | 3 | 3 |
| 10^-6^ M | 12 | 3 | 3 | 3 | 3 | 3 | 3 |
| 10^-5^ M | 12 | 3 | 3 | 3 | 3 | 3 | 3 |

Note: HK-2 cells were incubated with a mixture of ang-(1-7) and C21.

**Table 5.** Sample size (n) corresponding to the ligand combinations studied (as in Figure 5 of manuscript).

| AVE | | | | | | | |
| --- | --- | --- | --- | --- | --- | --- | --- |
| C21 | 0 M | 10^-10^ M | 10^-9^ M | 10^-8^ M | 10^-7^ M | 10^-6^ M | 10^-5^ M |
| 0 M |  | 6 | 5 | 6 | 6 | 8 | 8 |
| 10^-10^ M | 6 | 5 | 5 | 5 | 5 | 5 | 5 |
| 10^-9^ M | 6 | 5 | 5 | 5 | 5 | 5 | 5 |
| 10^-8^ M | 6 | 5 | 5 | 5 | 5 | 5 | 4 |
| 10^-7^ M | 8 | 5 | 5 | 3 | 5 | 5 | 5 |
| 10^-6^ M | 12 | 5 | 5 | 5 | 5 | 5 | 5 |
| 10^-5^ M | 12 | 4 | 4 | 4 | 4 | 4 | 4 |

Note: HK-2 cells were pre-incubated with AVE0991 followed by C21.

**Table 6.** Comparison of Bliss synergy score with that of ZIP synergy score.

| **Case**  HK-2 cells were incubated with | Bliss synergy | ZIP synergy |
| --- | --- | --- |
| **1.** ang-(1-7) followed by ang-II (Fig. 1 of manuscript) | 162 | -2 |
| **2.** ang-(1-7) followed by C21 (Fig. 2 of manuscript) | 304 | 26 |
| **3.** C21 followed by ang-(1-7) (Fig. 3 of manuscript) | 484 | 27 |
| **4.** a mixture of ang-(1-7) and C21 (Fig. 4 of manuscript) | 76 | 20 |
| **5.** AVE0991 followed by C21 (Fig. 5 of manuscript) | 45 | 15 |





**Figure 1.** Effect of ang-(1-7), C21, AVE and ang-II on formation of nitric oxide in HK-2 cells. These responses are same as represented in Figures. 1-5 in original manuscript as responses of ligand alone.


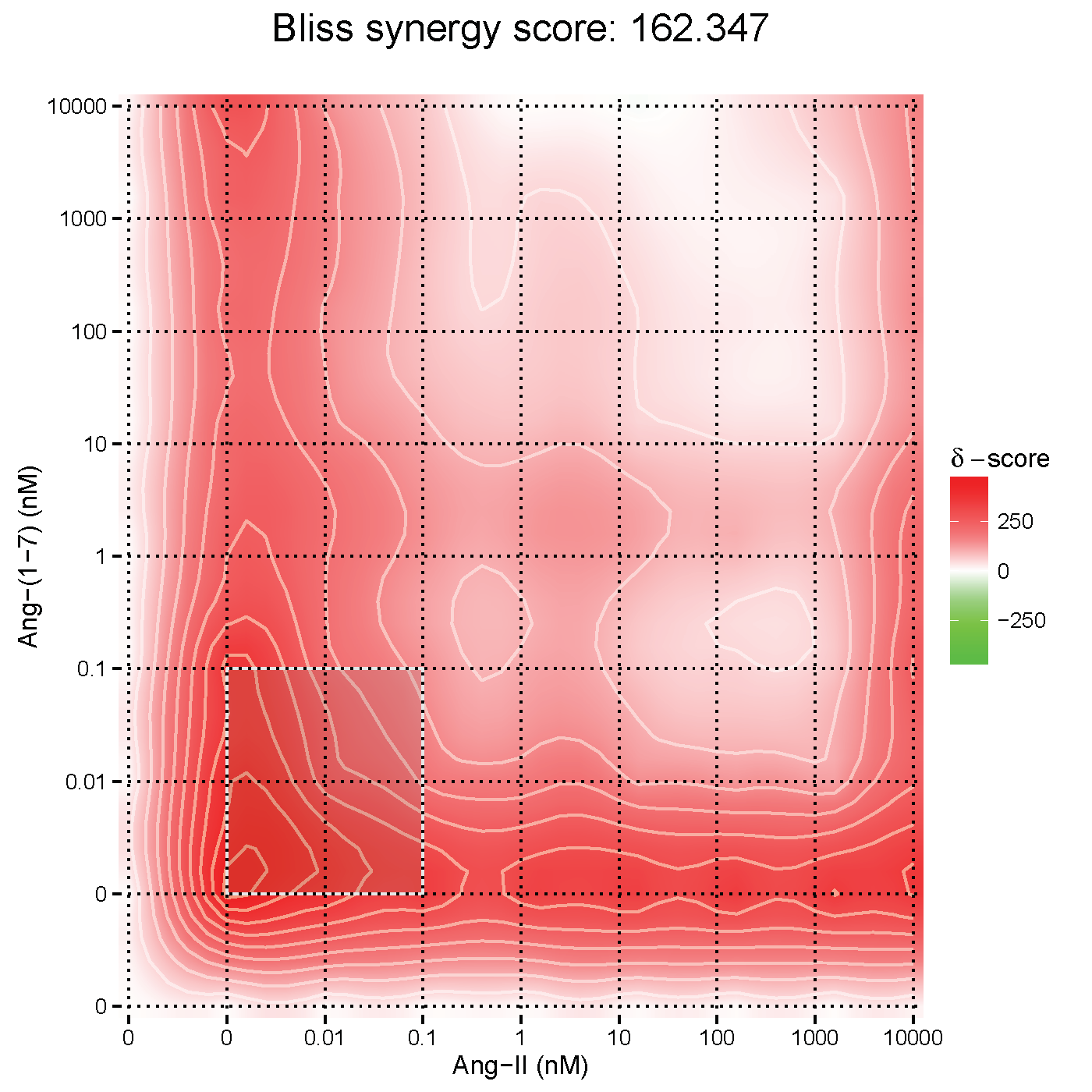


**Figure 2.** The 2-D plot showing **Bliss** synergistic spots while HK-2 cells pre-incubated with ang-(1-7) followed by ang-II supporting the results of Figure 1 of manuscript.

**A**


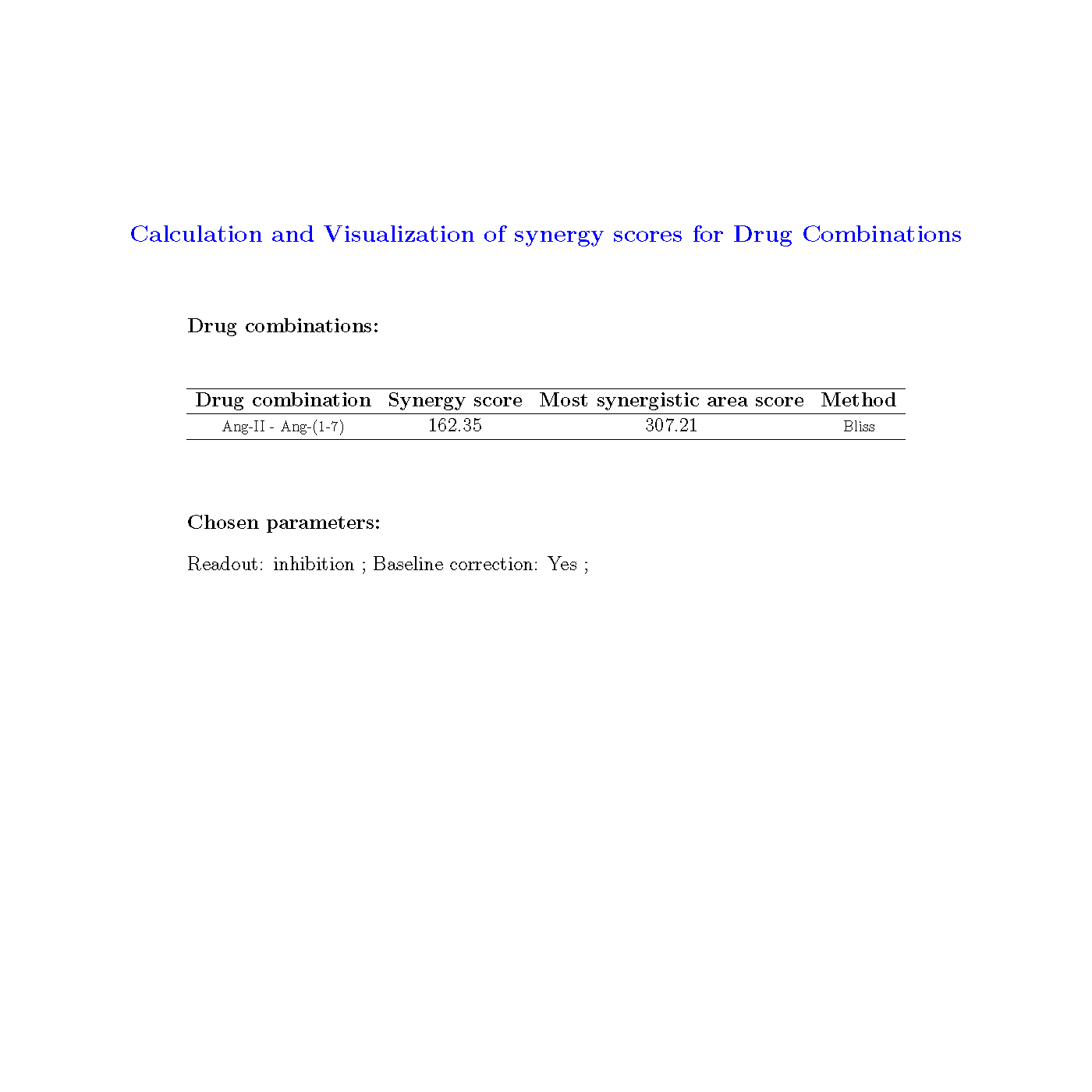


**B**


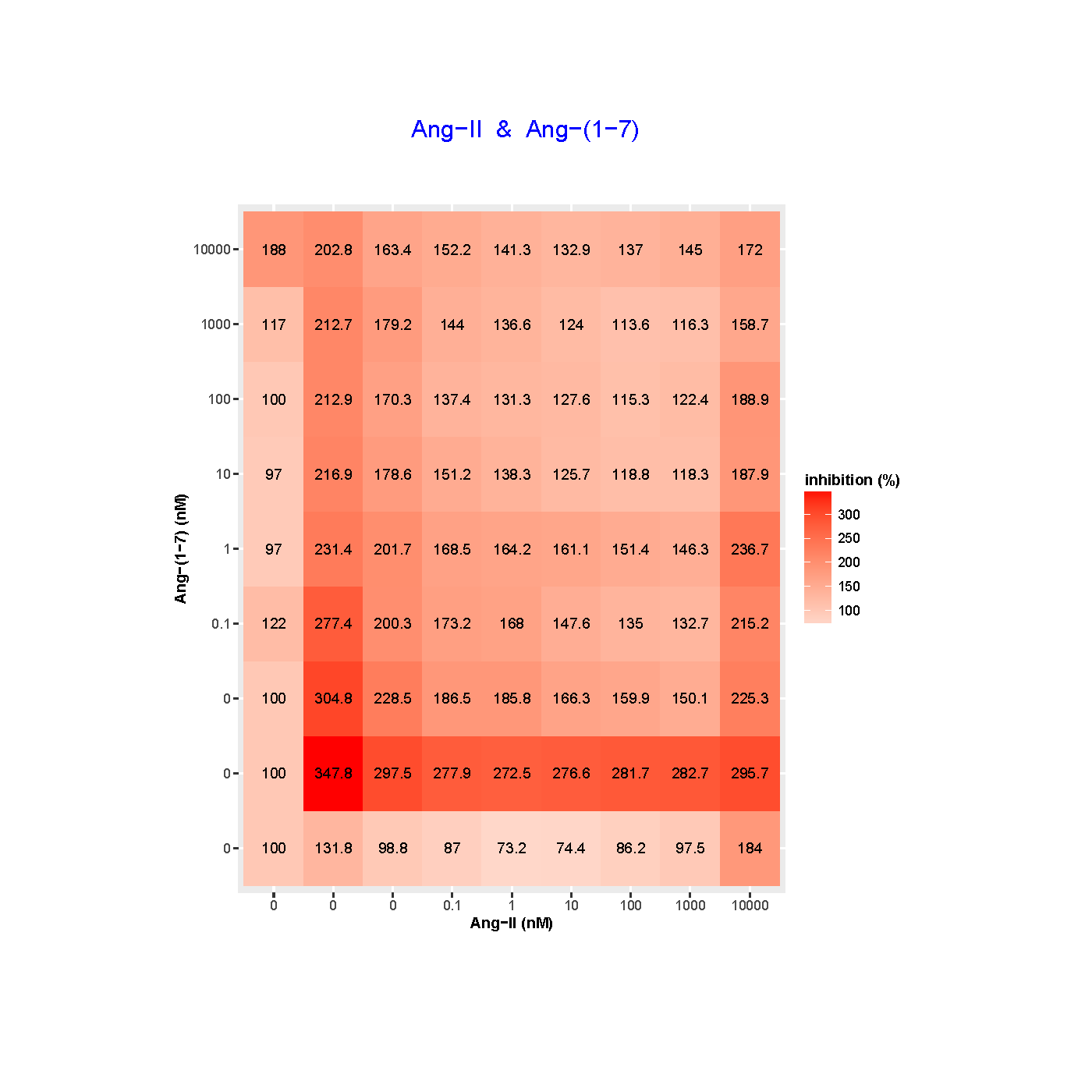


**Figure 3.** The calculation and visualization report of **Bliss** synergy score derived from SynergyFinder (version 2.0) (a) and matrix results for various combination of ligands (b) while HK-2 cells pre-incubated with ang-(1-7) followed by ang-II supporting the results of Figure 1 of manuscript.


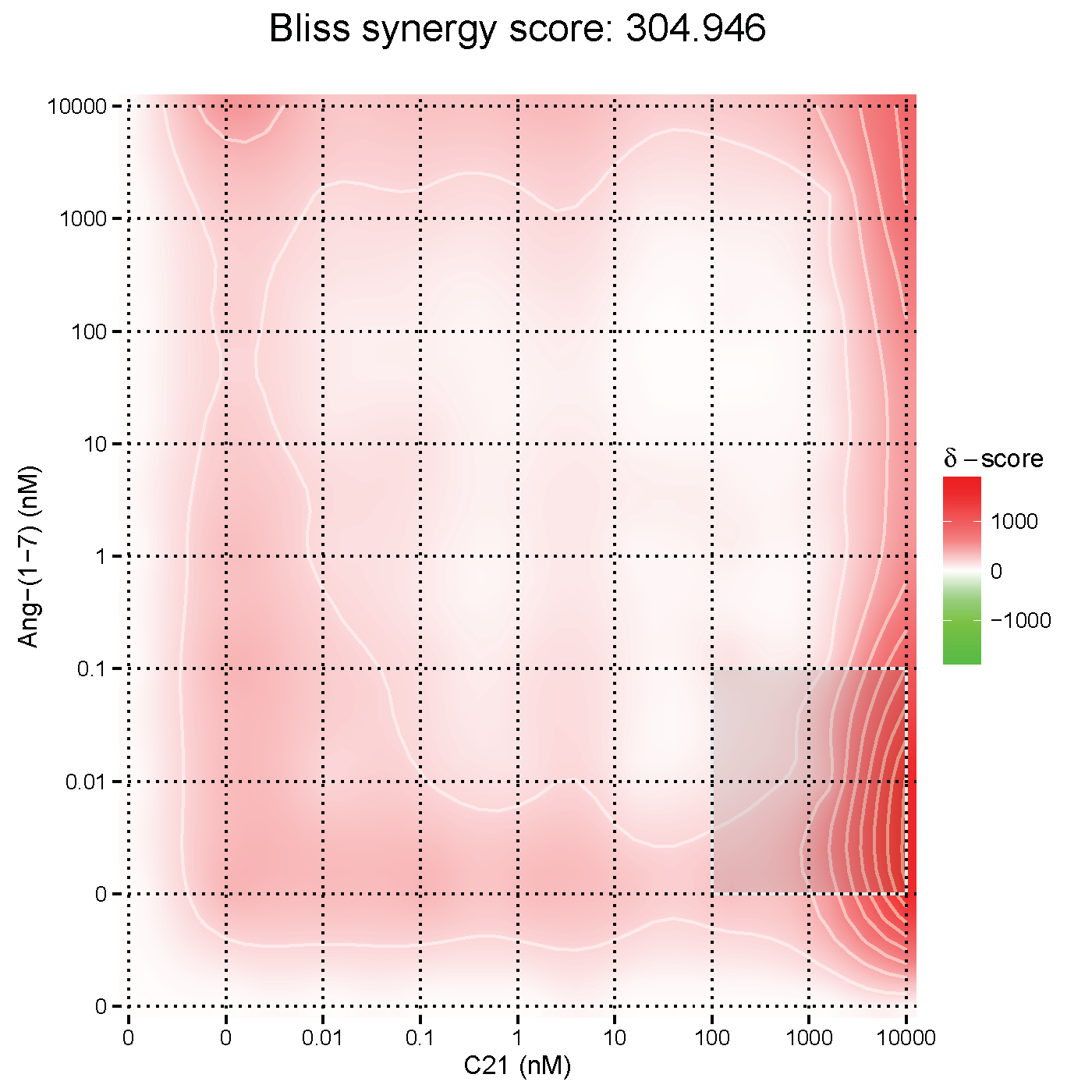


**Figure 4.** The 2-D plot showing **Bliss** synergistic spots while HK-2 cells pre-incubated with ang-(1-7) followed by C21 supporting the results of Figure 2 of manuscript.

**A**


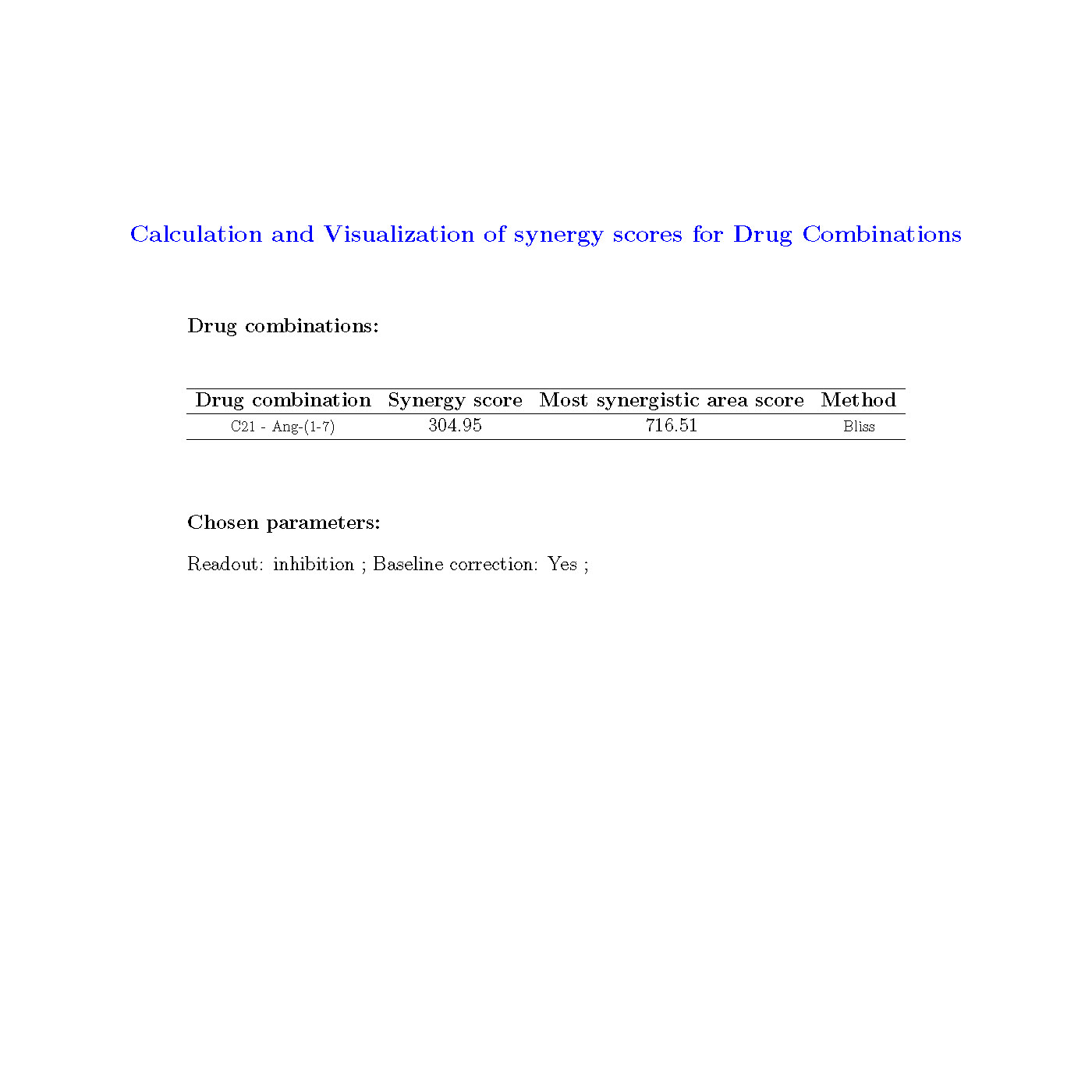


**B**

**
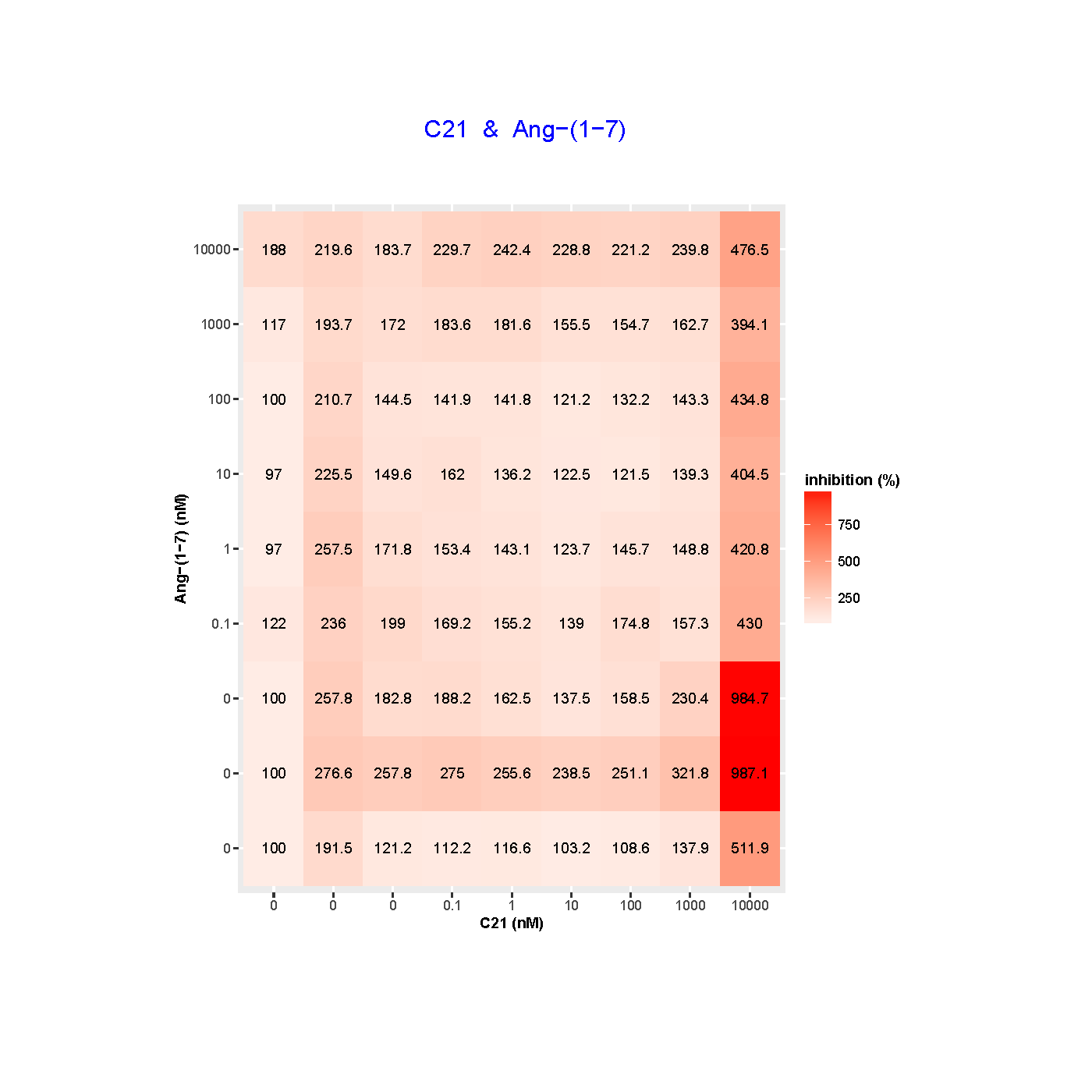
**

**Figure 5.** The calculation and visualization report of **Bliss** synergy score derived from SynergyFinder (version 2.0) (a) and matrix results for various combination of ligands (b) while HK-2 cells pre-incubated with ang-(1-7) followed by C21 supporting the results of Figure 2 of manuscript.


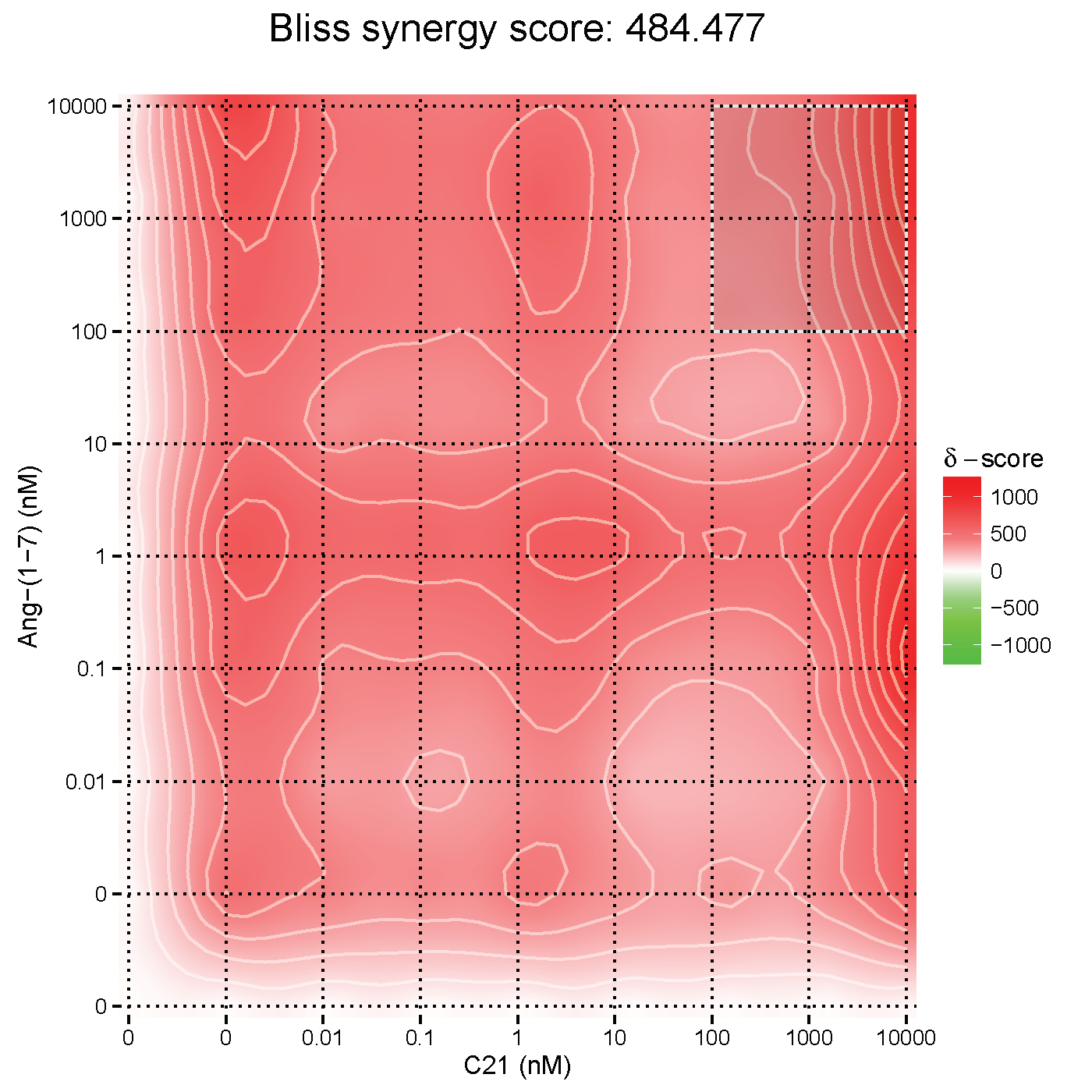


**Figure 6.** The 2-D plot showing **Bliss** synergistic spots while HK-2 cells pre-incubated with C21 followed by ang-(1-7) supporting the results of Figure 3 of manuscript.

**A**


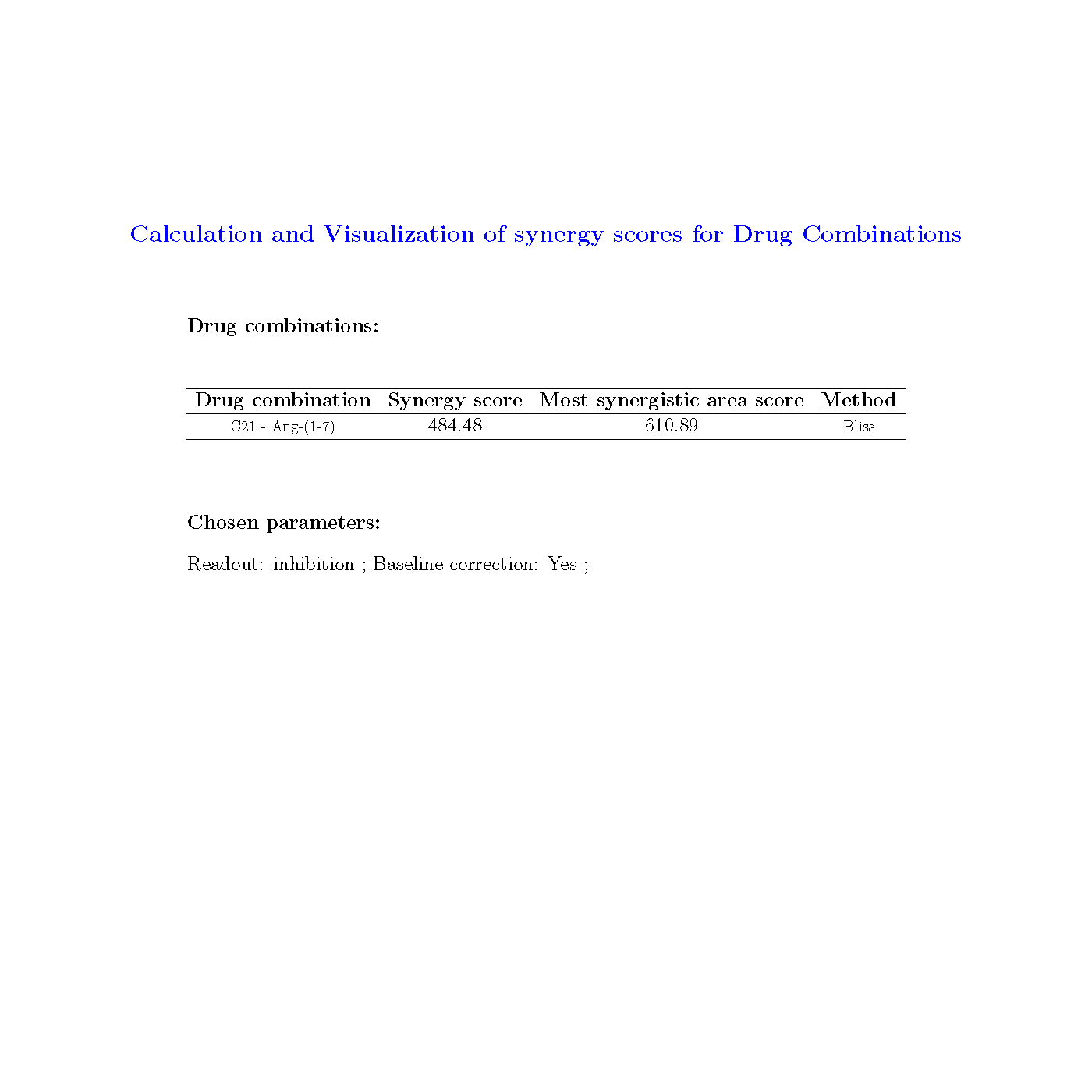


**B**


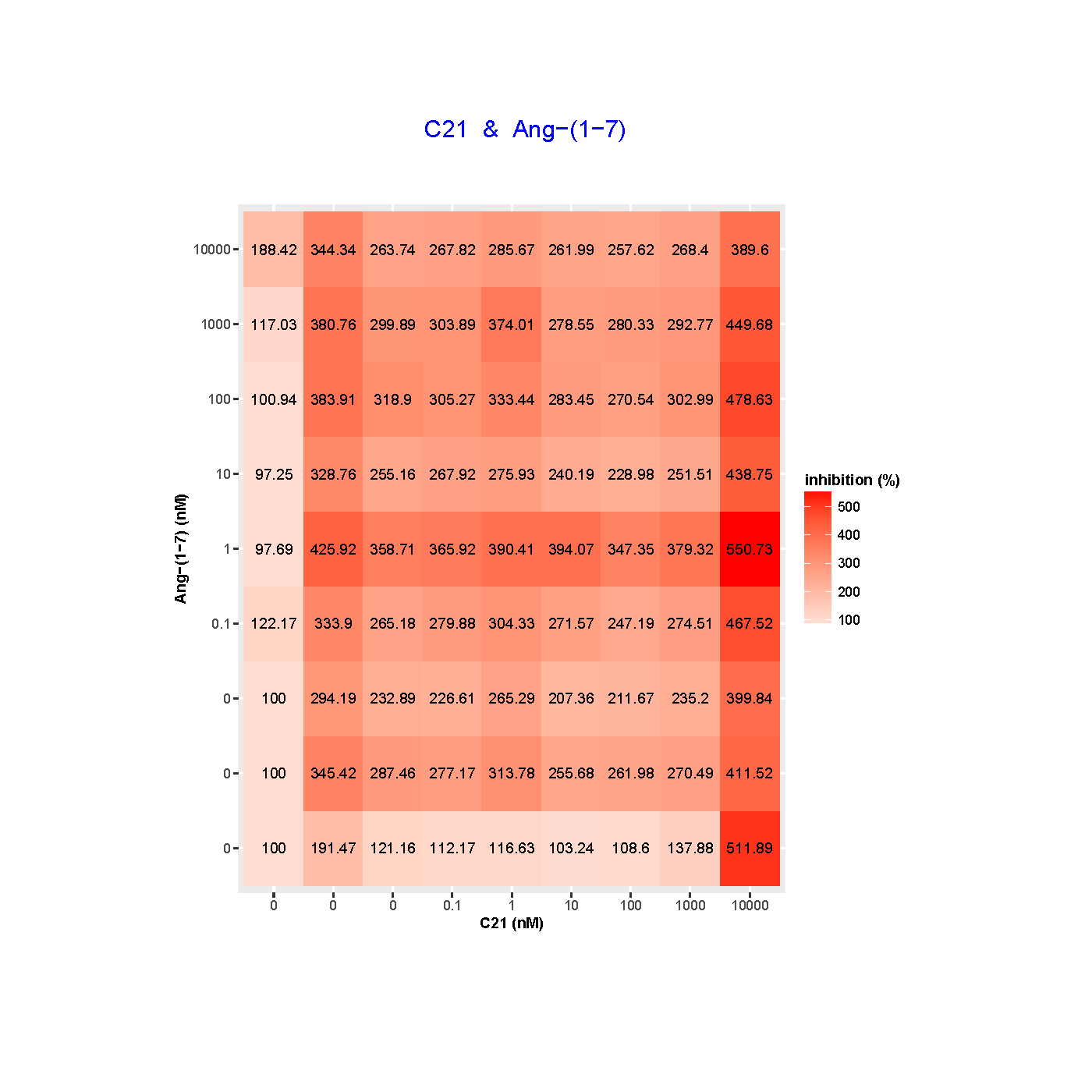


**Figure 7.** The calculation and visualization report of **Bliss** synergy score derived from SynergyFinder (version 2.0) (a) and matrix results for various combination of ligands (b) while HK-2 cells pre-incubated with C21 followed by ang-(1-7) supporting the results of Figure 3 of manuscript.


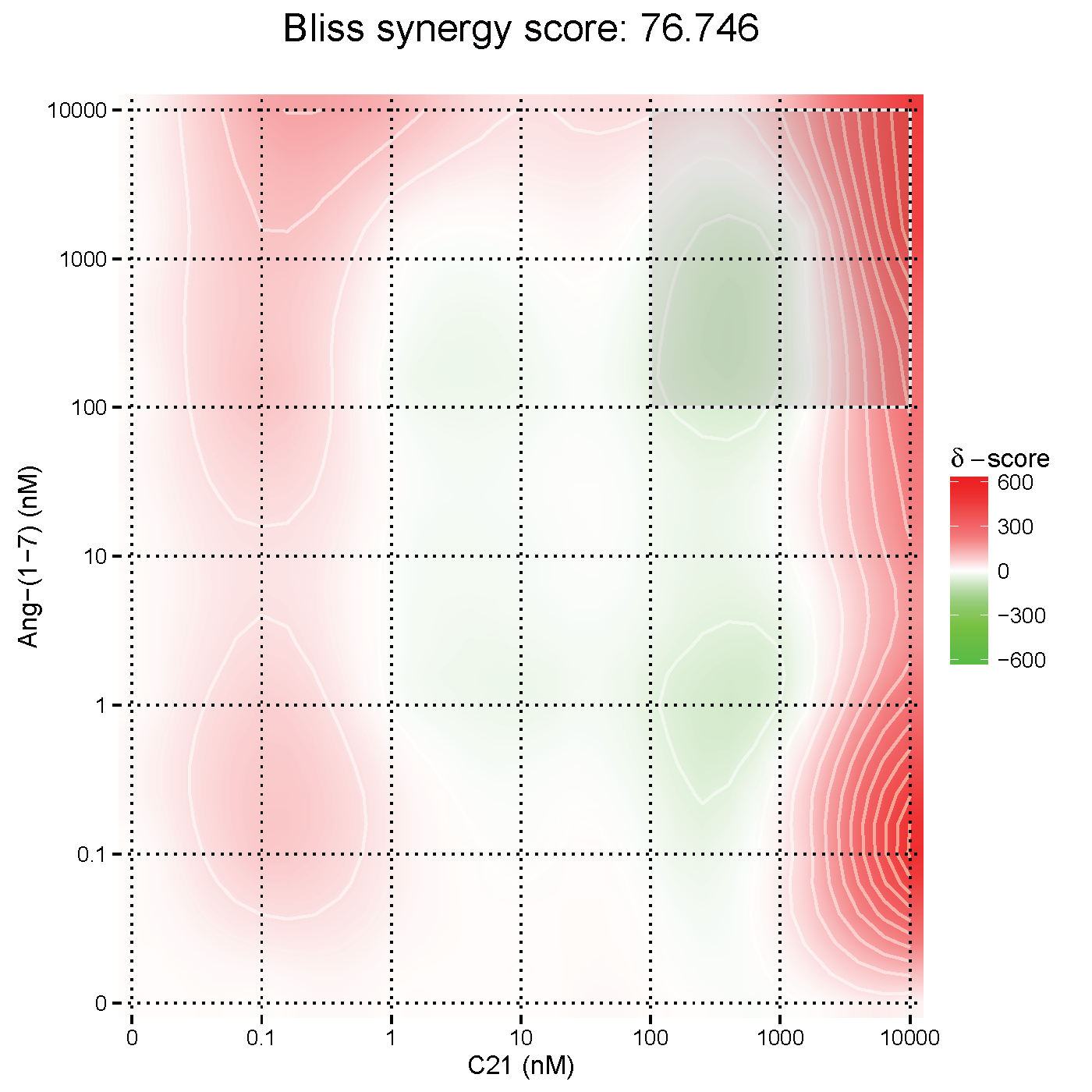


**Figure 8.** The 2-D plot showing **Bliss** synergistic spots while HK-2 cells incubated with a mixture of ang-(1-7) and C21 supporting the results of Figure 4 of manuscript.

**A**


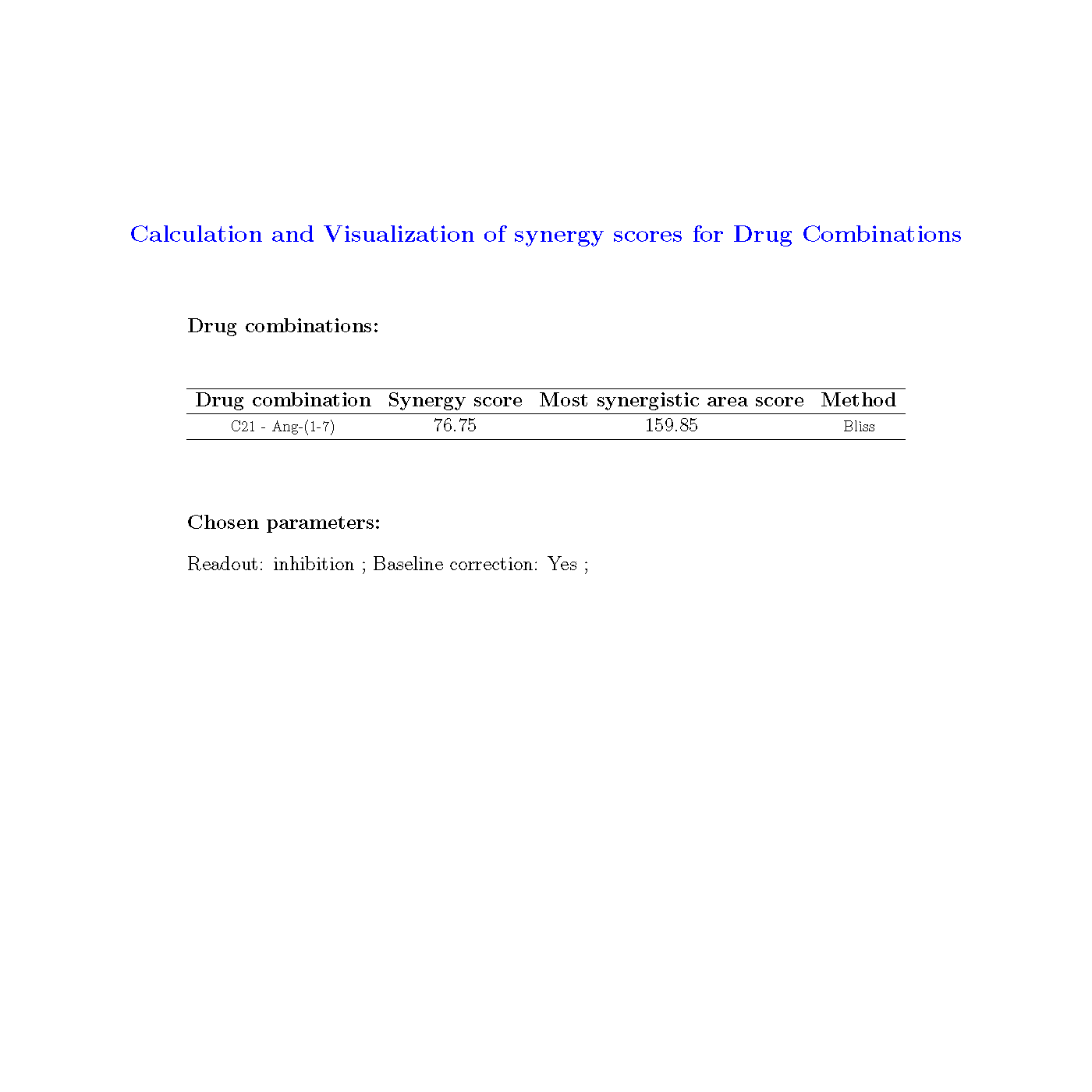


**B**


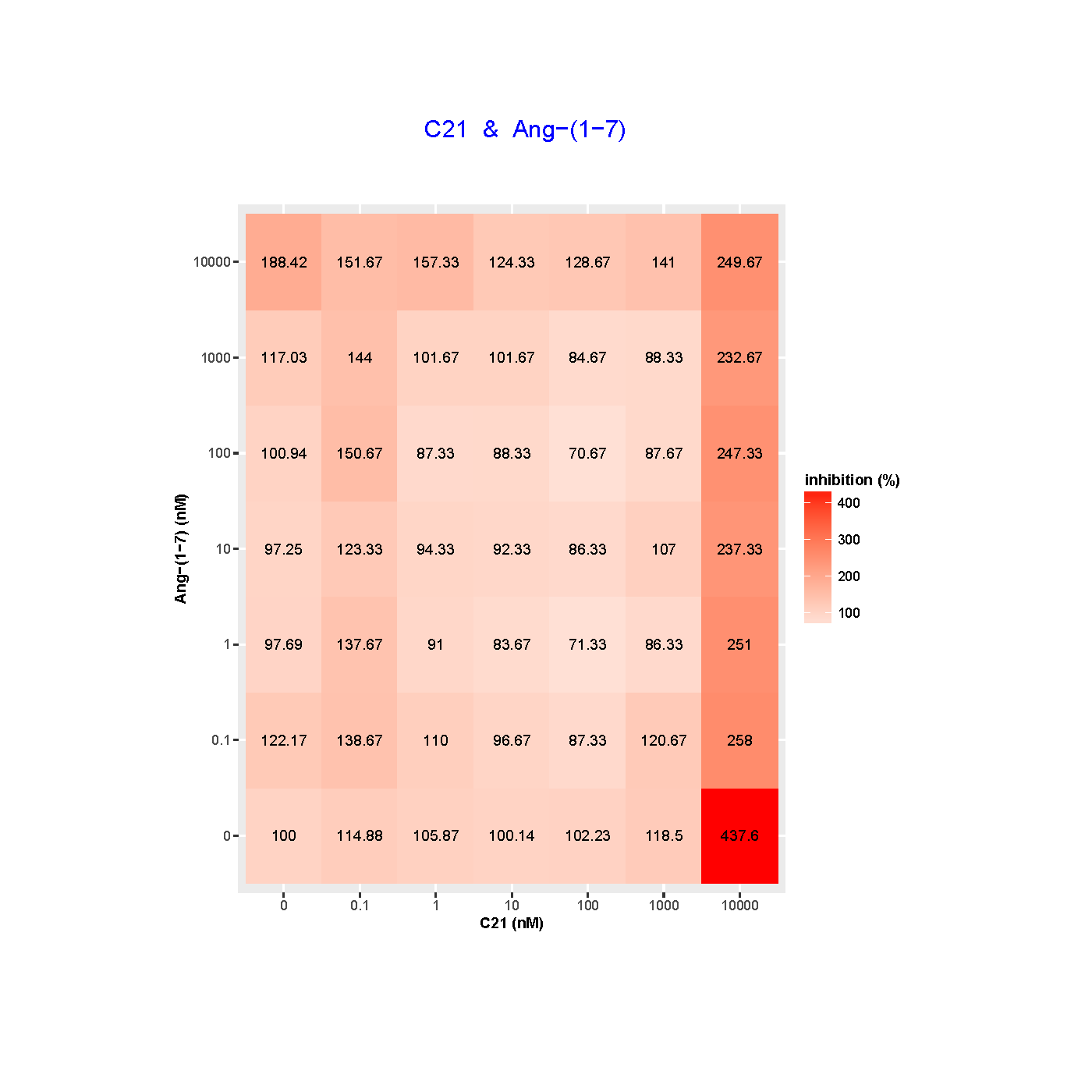


**Figure 9.** The calculation and visualization report of **Bliss** synergy score derived from SynergyFinder (version 2.0) (a) and matrix results for various combination of ligands (b) while HK-2 cells incubated with a mixture of ang-(1-7) and C21 supporting the results of Figure 4 of manuscript.


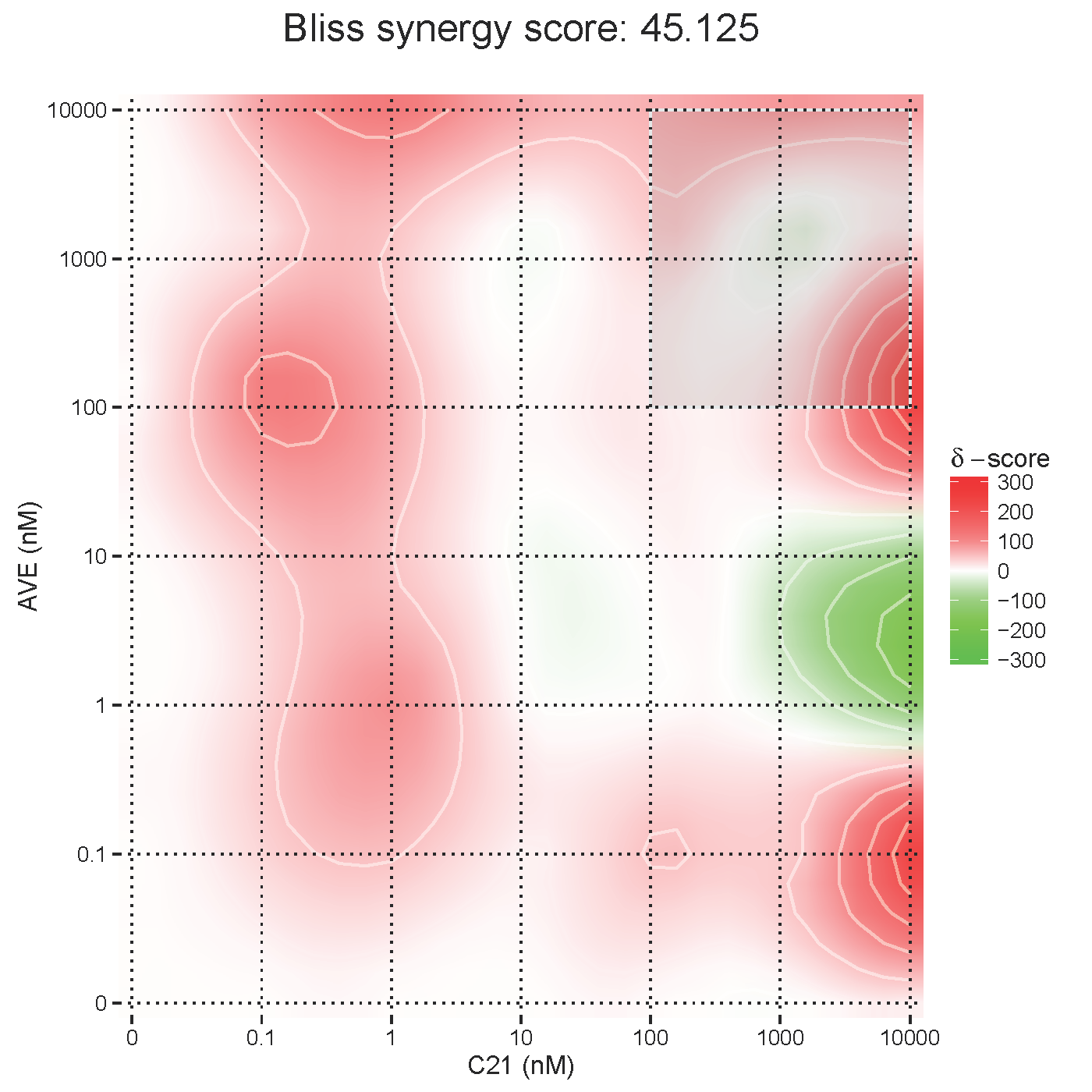


**Figure 10.** The 2-D plot showing **Bliss** synergistic spots while HK-2 cells pre-incubated with AVE followed by C21 supporting the results of Figure 5 of manuscript.

**A**


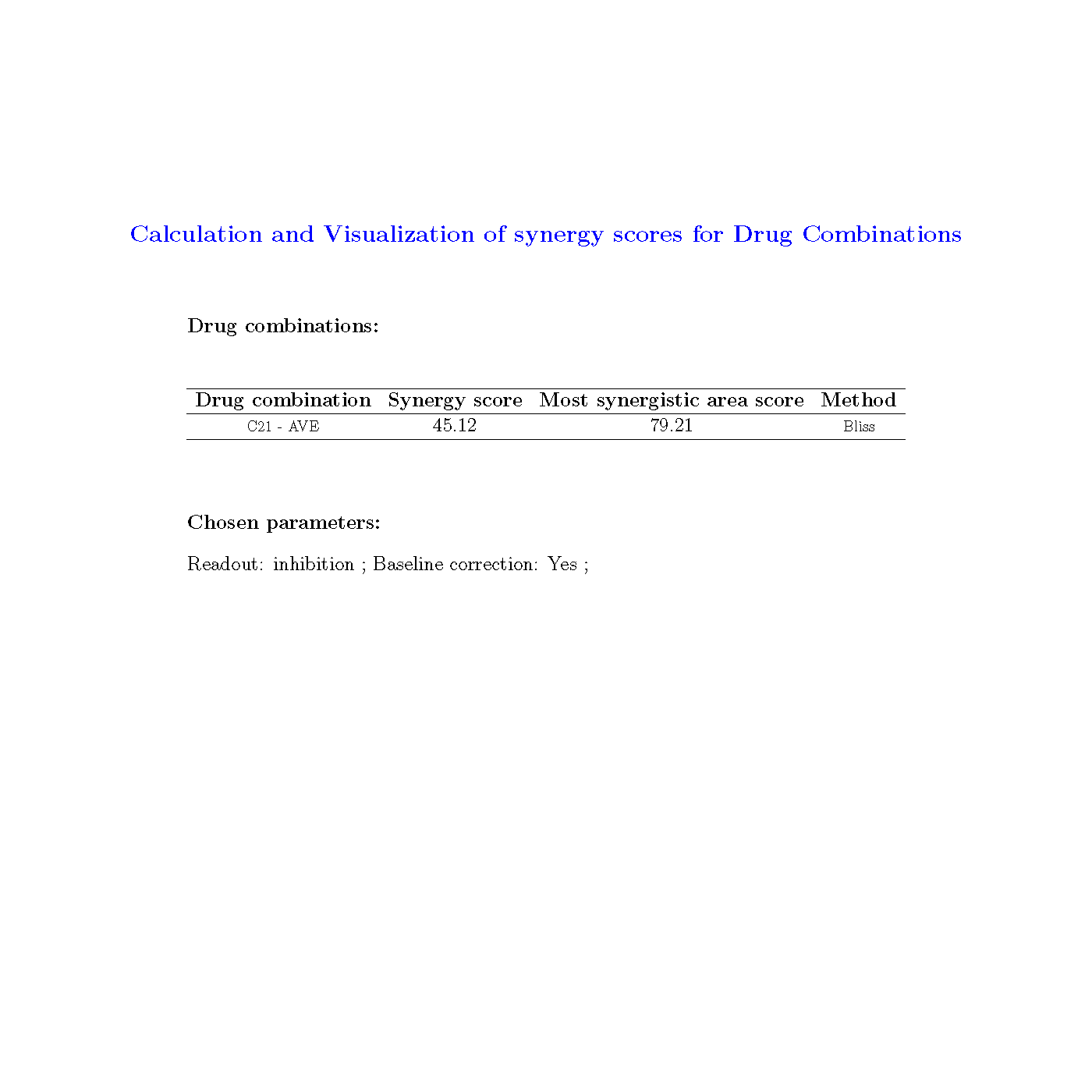


**B**


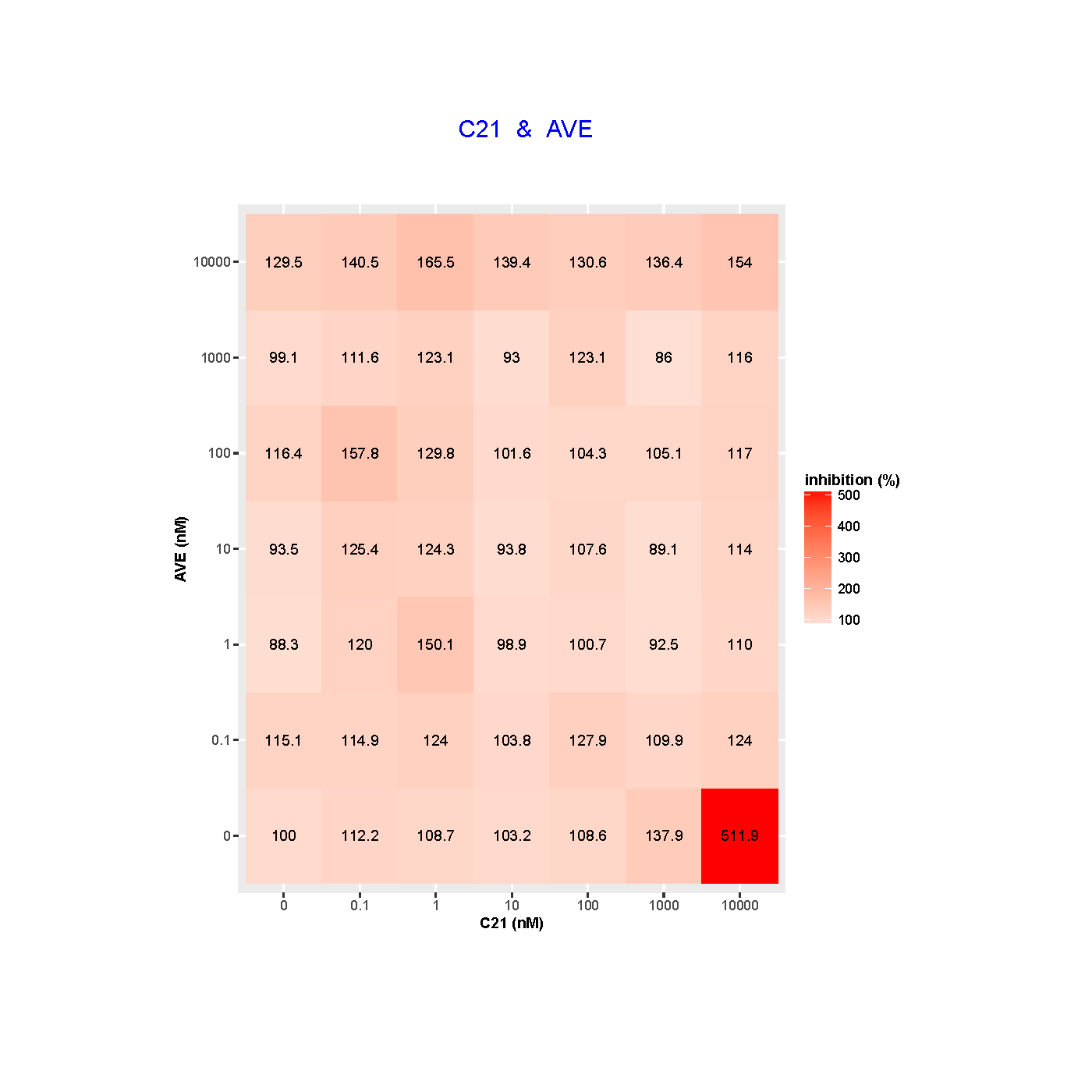


**Figure 11.** The calculation and visualization report of **Bliss** synergy score derived from SynergyFinder (version 2.0) (a) and matrix results for various combination of ligands (b) while HK-2 cells pre-incubated with AVE followed by C21 supporting the results of Figure 5 of manuscript.


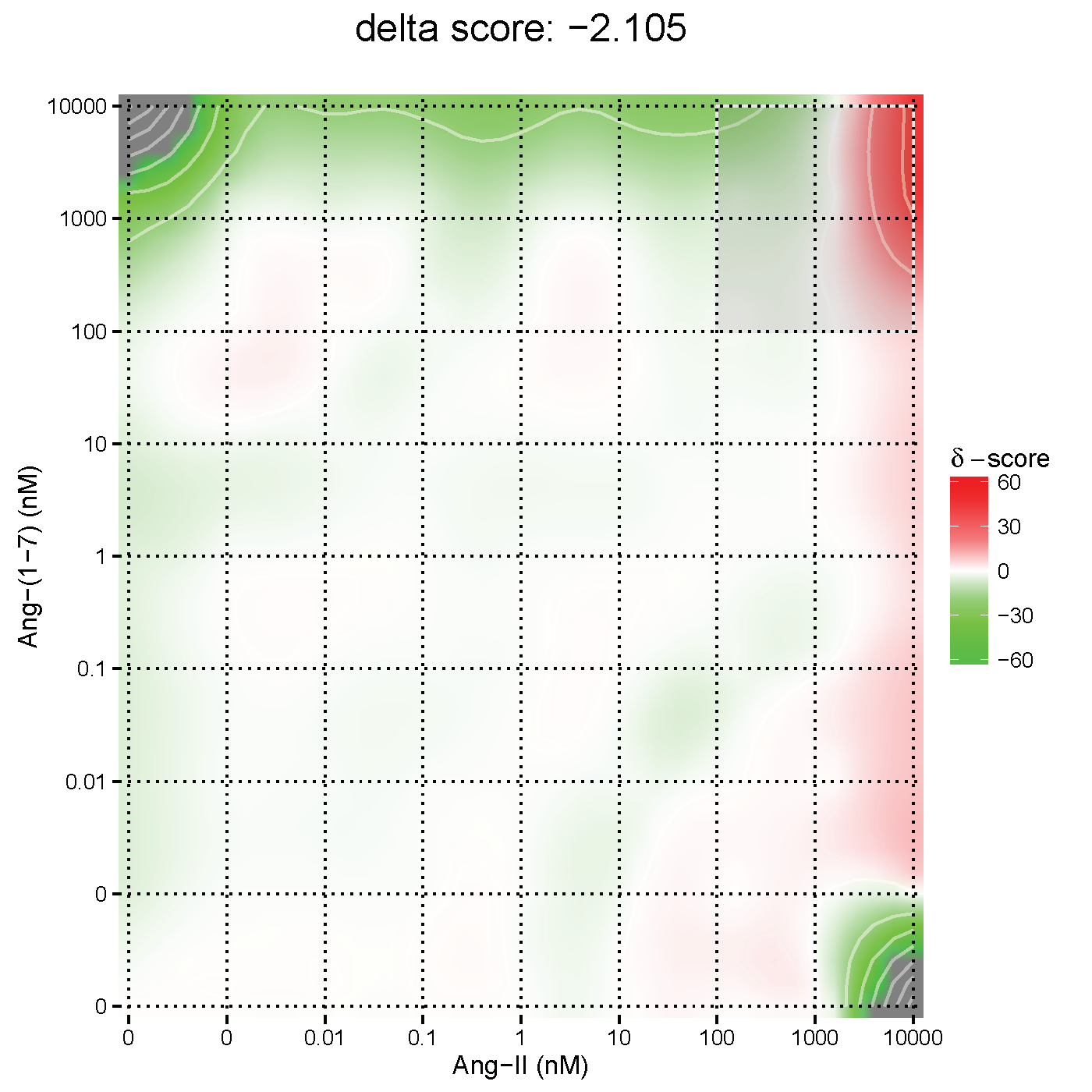


**Figure 12.** The 2-D plot showing **ZIP** synergistic spots while HK-2 cells pre-incubated with ang-(1-7) followed by ang-II supporting the results of Figure 1 of manuscript.


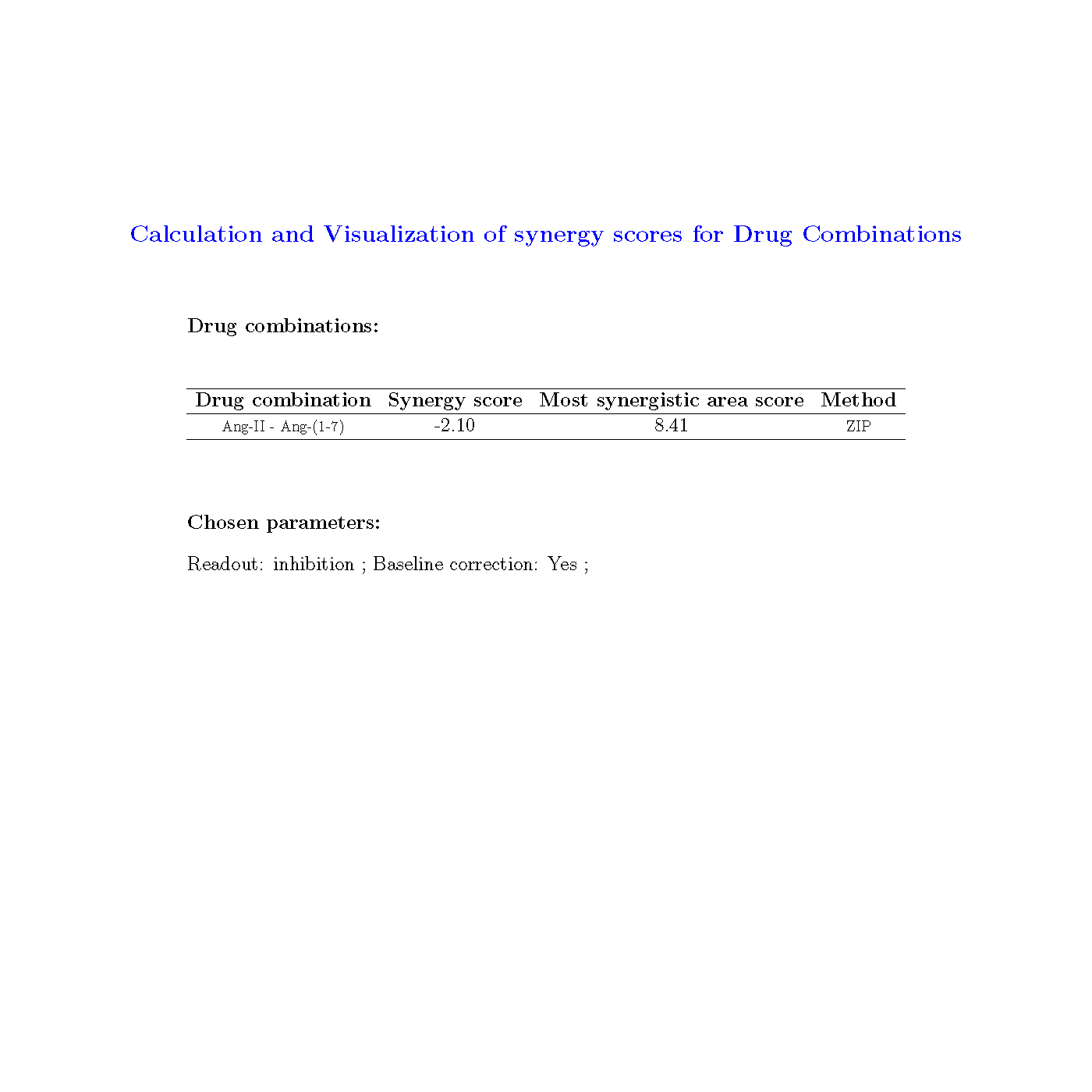


**Figure 13.** The calculation and visualization report of **ZIP** synergy score derived from SynergyFinder (version 2.0) while HK-2 cells pre-incubated with ang-(1-7) followed by ang-II supporting the results of Figure 1 of manuscript.


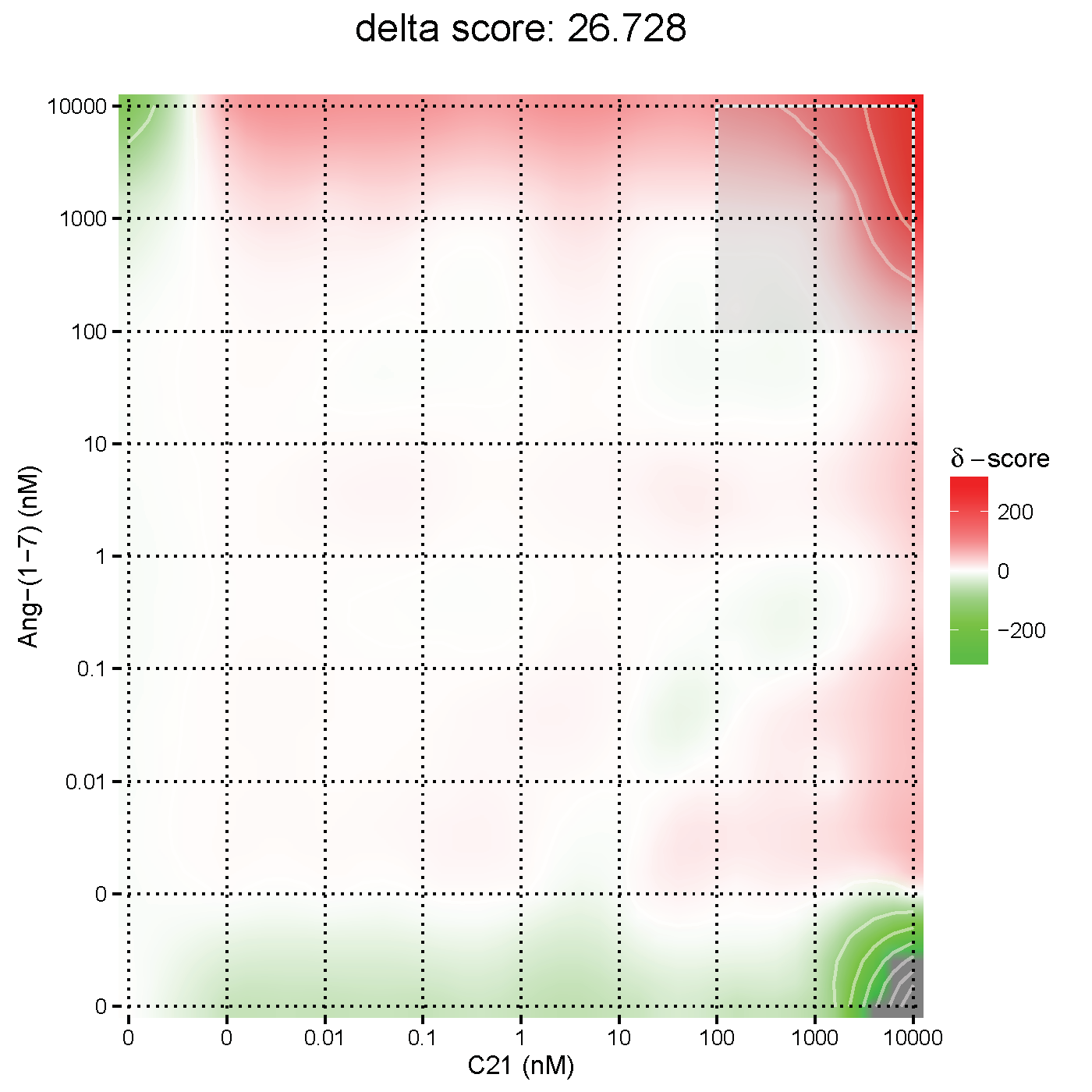


**Figure 14.** The 2-D plot showing **ZIP** synergistic spots while HK-2 cells pre-incubated with ang-(1-7) followed by C21 supporting the results of Figure 2 of manuscript.

**
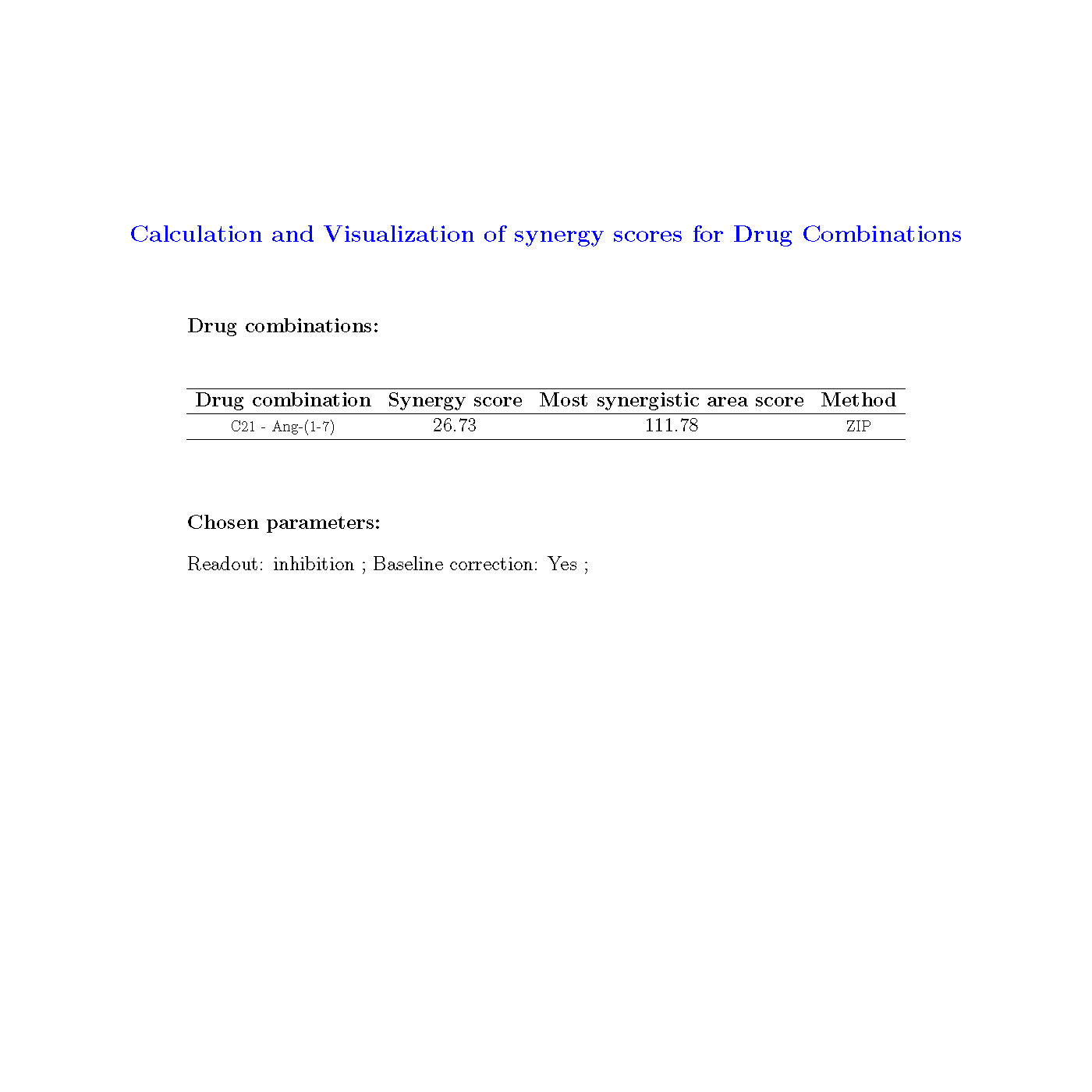
**

**Figure 15.** The calculation and visualization report of **ZIP** synergy score derived from SynergyFinder (version 2.0) while HK-2 cells pre-incubated with ang-(1-7) followed by C21 supporting the results of Figure 2 of manuscript.


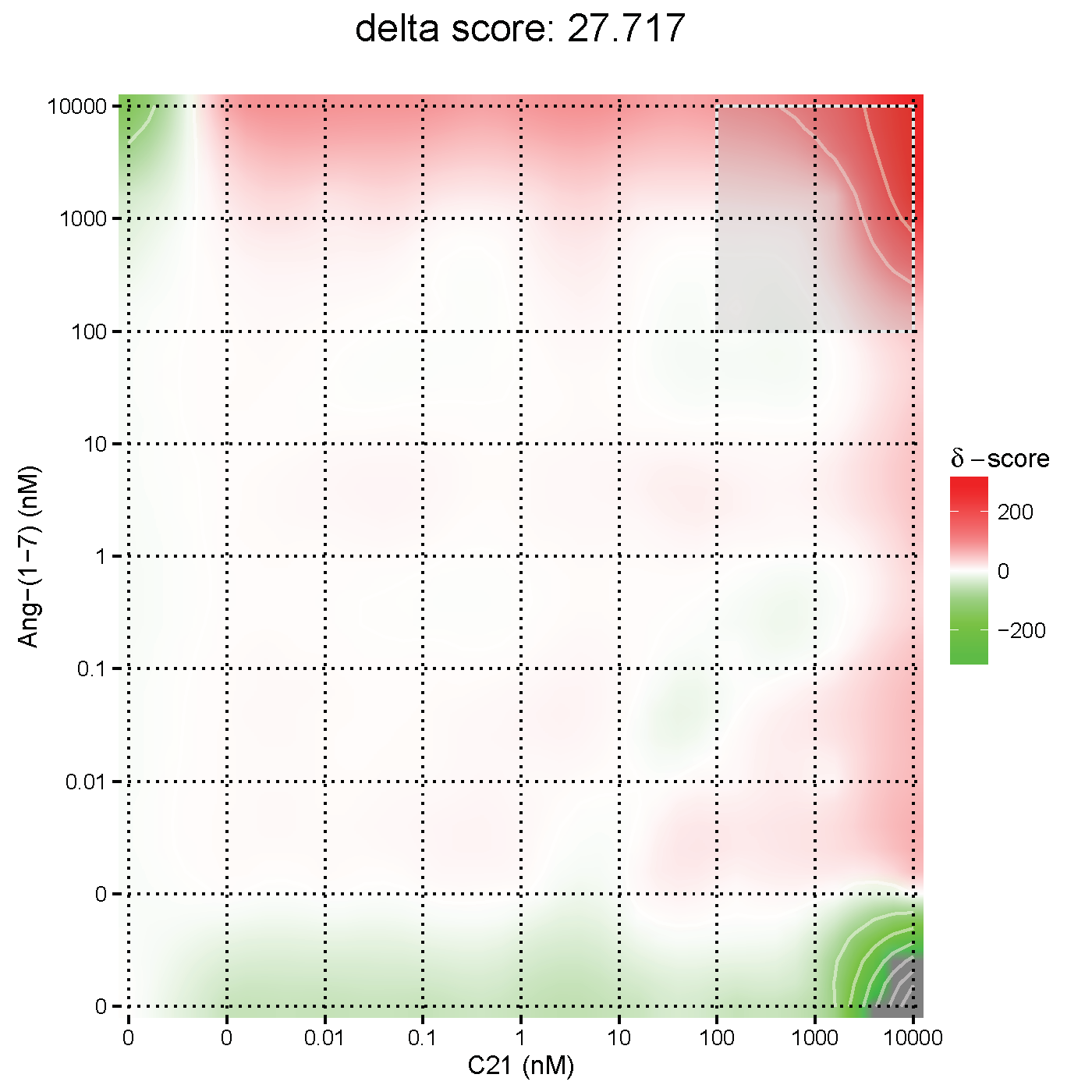


**Figure 16.** The 2-D plot showing **ZIP** synergistic spots while HK-2 cells pre-incubated with C21 followed by ang-(1-7) supporting the results of Figure 3 of manuscript.

**
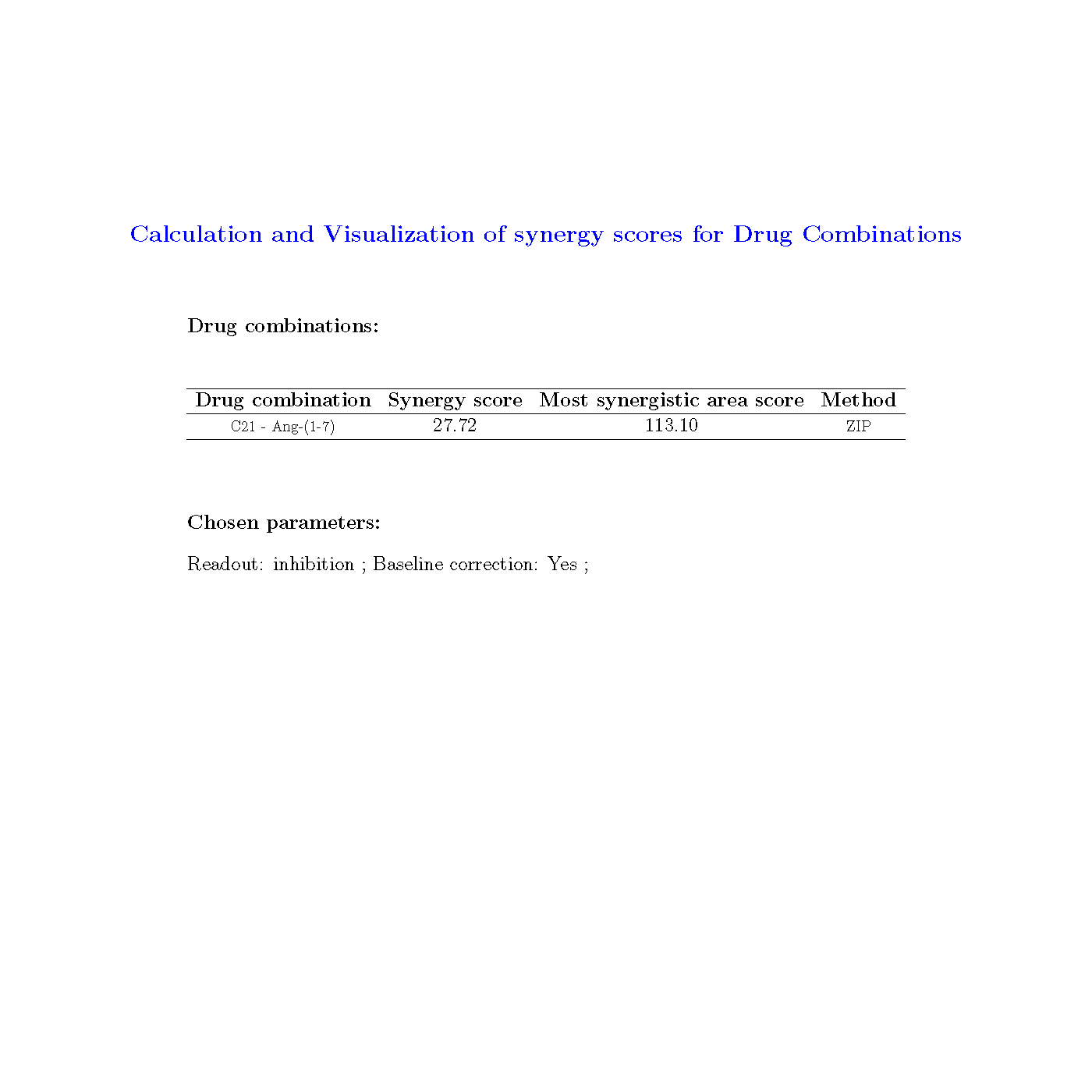
**

**Figure 17.** The calculation and visualization report of **ZIP** synergy score derived from SynergyFinder (version 2.0) while HK-2 cells pre-incubated with C21 followed by ang-(1-7) supporting the results of Figure 3 of manuscript.


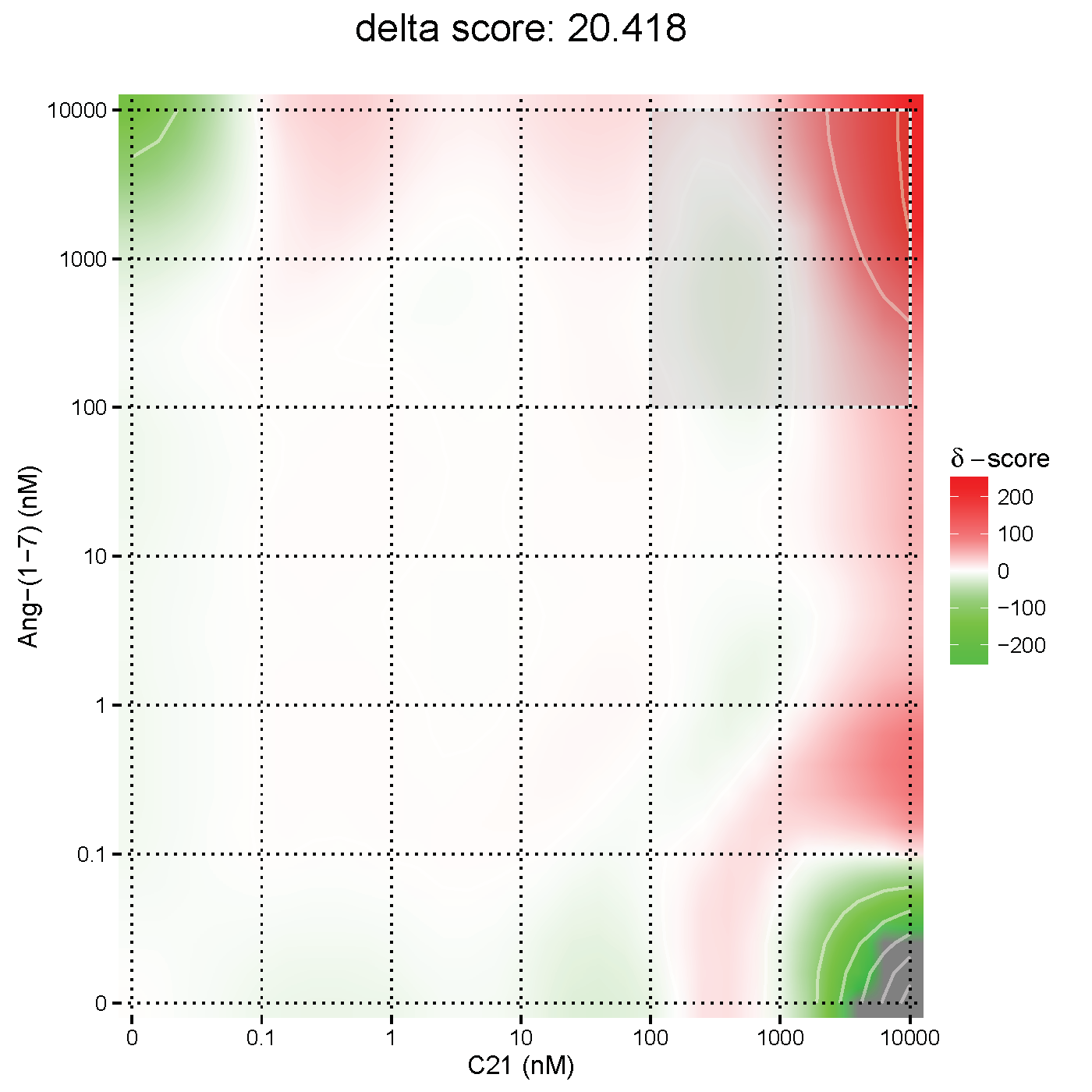


**Figure 18.** The 2-D plot showing **ZIP** synergistic spots while HK-2 cells incubated with a mixture of ang-(1-7) and C21 supporting the results of Figure 4 of manuscript.

**
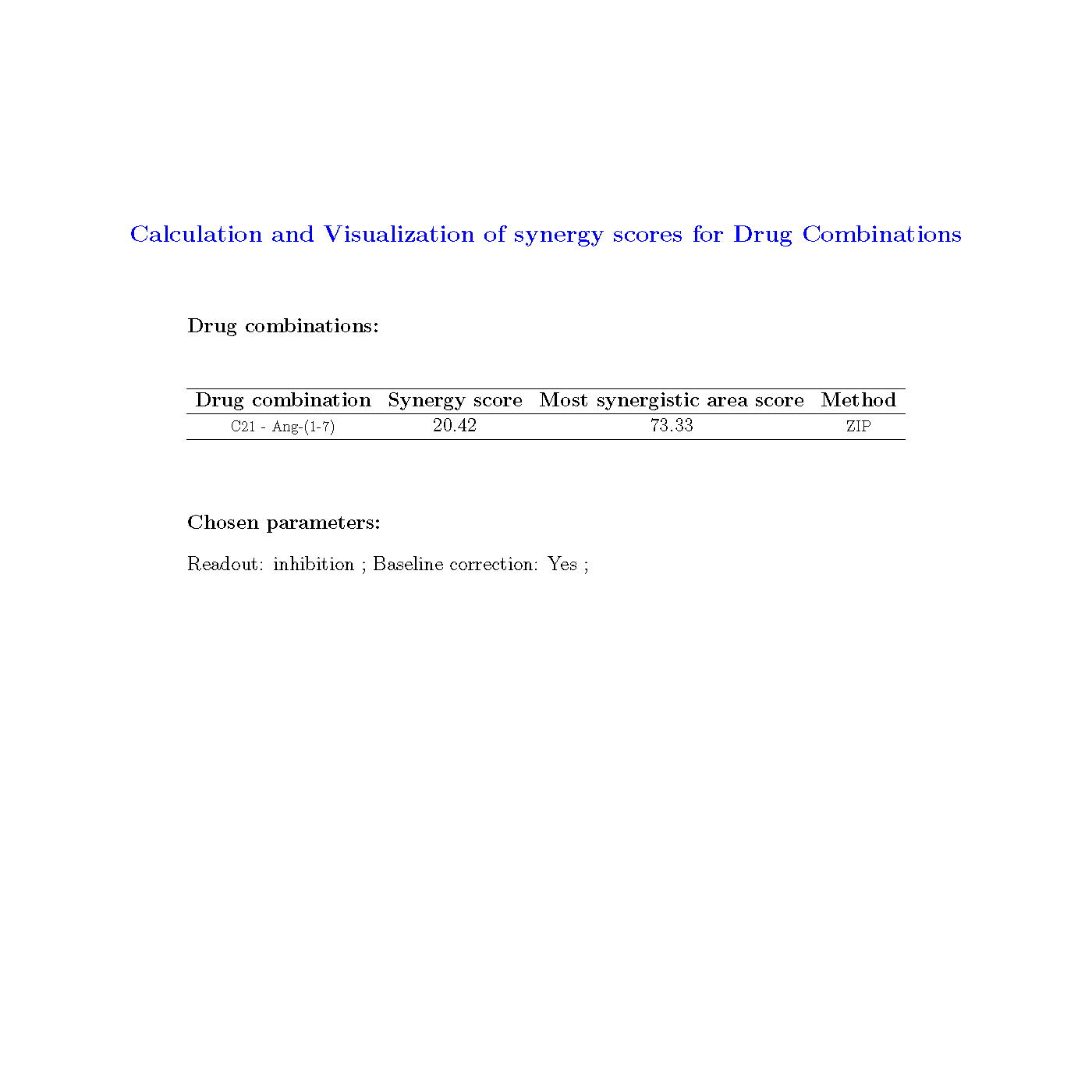
**

**Figure 19.** The calculation and visualization report of **ZIP** synergy score derived from SynergyFinder (version 2.0) while HK-2 cells incubated with a mixture of ang-(1-7) and C21 supporting the results of Figure 4 of manuscript.


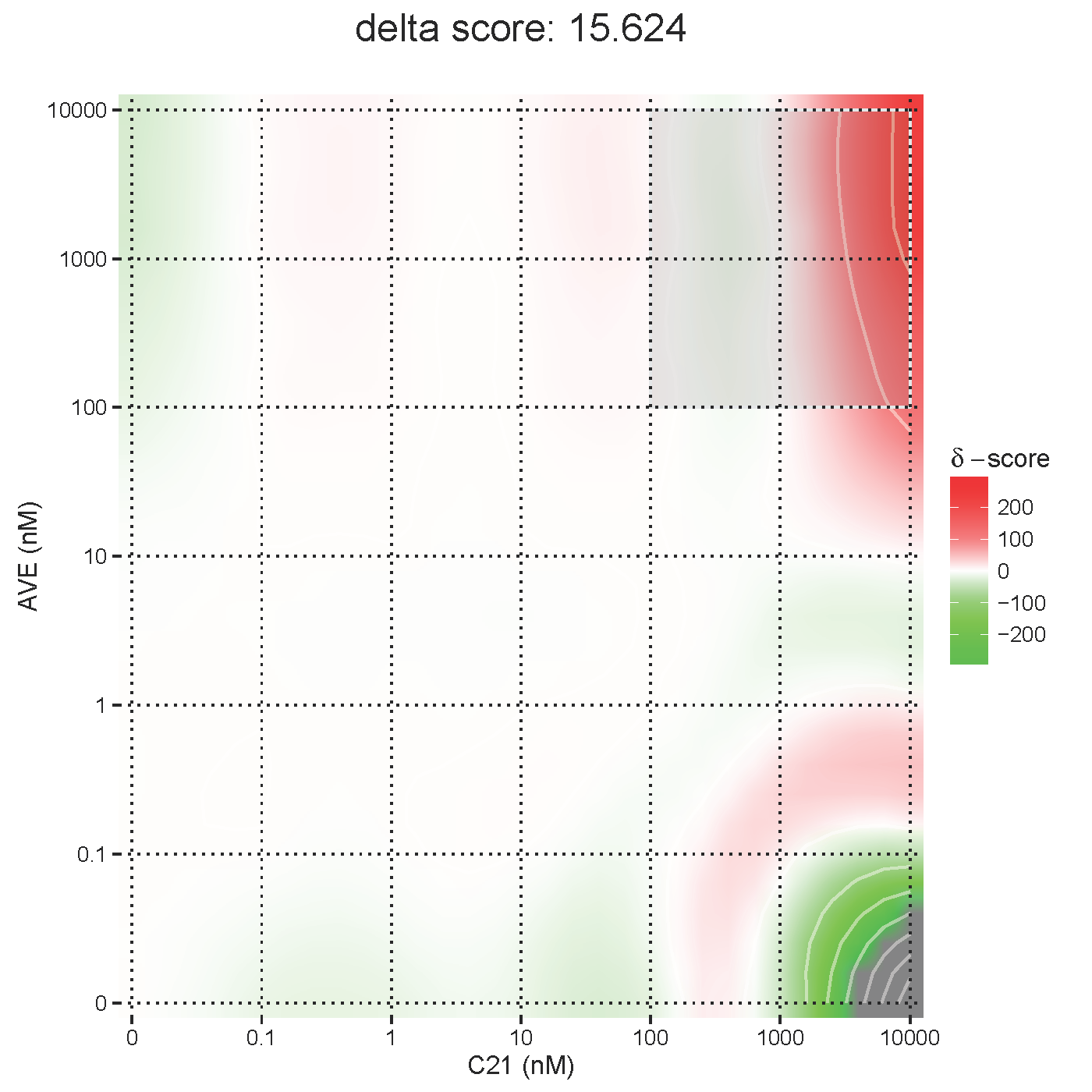


**Figure 20.** The 2-D plot showing **ZIP** synergistic spots while HK-2 cells pre-incubated with AVE followed by C21 supporting the results of Figure 5 of manuscript.

**
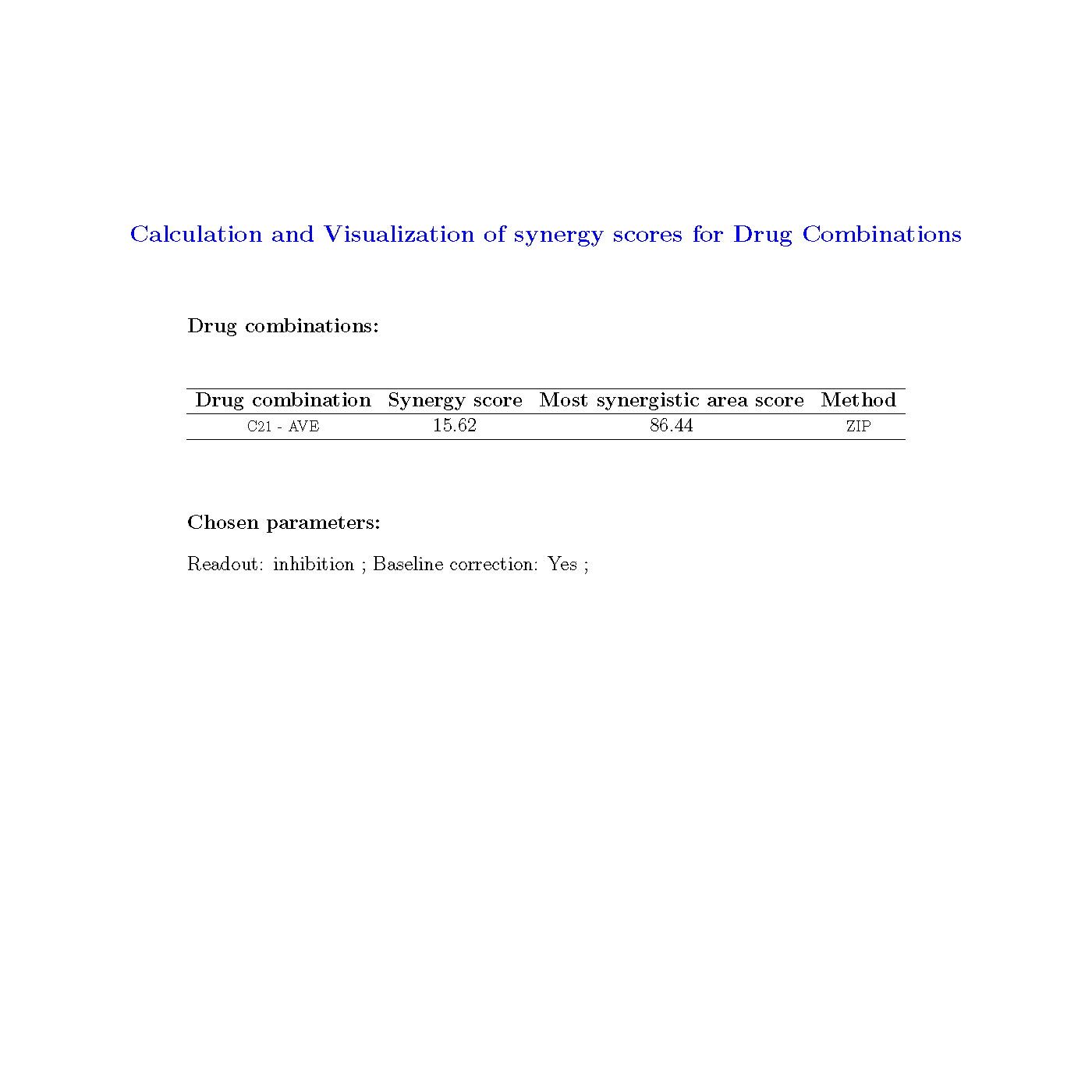
**

**Figure 21.** The calculation and visualization report of **Bliss** synergy score derived from SynergyFinder (version 2.0) while HK-2 cells pre-incubated with AVE followed by C21 supporting the results of Figure 5 of manuscript.

**

Figure 22.** The representation of general theorem of combination index (log[CI]) (a) and dose(concentration)-reduction index (log[DRI]) (b) allowing quantitative determination of agonist interactions according to Chou and Talalay [[1](#_ENREF_1),[2](#_ENREF_2)]. The CI and DRI are represented on log-scale for the inclusion of out-of-scale points. The log[CI] values at any given effect-size (Fa) were derived from actual data points (fold change) in the concentration-response non-linear regression curves. Combinations are additive at log[CI]=0, synergistic at log[CI]<0 and antagonistic at log[CI]>0. The line of additivity crossing at zero is shown. The log[DRI] plot at any given effect-size level (Fa) is favourable where log[DRI]>0 in case of synergy.





**Figure 23.** The reduction in log[CI] (a,c) and an increase in log[DRI] (b,d) shows synergistic formation of nitric oxide upon pre-incubation of HK-2 cells with ang-(1-7) followed by addition of C21. The “variable concentration” method was used for analysis of nitric oxide response of ang-(1-7) and C21 in HK-2 cells. Cells were stimulated with a variable serially diluted concentration of ang-(1-7) (10^-10^-10^-7^ M) for 10 minute plus C21 (serial dilution of 10^-10^-10^-7^ M). Results were fitted to a four-parametric non-linear regression model using CompuSyn software (ComboSyn, Inc.). The, combination index (CI) and dose (concentration)-reduction index (DRI) values were derived based on median-effect equation and fractional product method at five different effect-size/fraction affected (Fa) levels (Fa=0.25, 0.5, 0.75, 0.9 and 0.97) that were achieved with single ligand alone or in combinations. The r value, i.e. the linear correlation coefficient of the median-effect plot signifying conformity or goodness of fit of the experimental data and only data points corresponding r>0.99 were considered for CI and DRI determination. The CI values were presented in log[CI]-Fa form (fold increase in nitric oxide production) to determine whether the interactions were synergistic (if log[CI]<0), additive (if log[CI]=0) or antagonistic (if log[CI]>0). The DRI values for ang-(1-7) and C21 were represented in the form of log(DRI)-Fa plot with log(DRI)>0 is considered a favorable reduction of ligand concentration when used in combination to exert a desirable effect.

**REFERENCES**

1. Chou TC. Drug combination studies and their synergy quantification using the Chou-Talalay method. *Cancer Res*. 2010;70:440-446. doi:10.1158/0008-5472.CAN-09-1947.

2. Chou TC. Theoretical basis, experimental design, and computerized simulation of synergism and antagonism in drug combination studies. *Pharmacological reviews*. 2006;58:621-681. doi:10.1124/pr.58.3.10.
